# Supplementary material for: YAP Circular RNA, circYap, Attenuates Cardiac Fibrosis via Binding with Tropomyosin-4 and Gamma-Actin Decreasing Actin Polymerization
Source: Mol Ther. 2020 Dec 3;29(3):1138–50. doi: 10.1016/j.ymthe.2020.12.004 (PMC7934790; doi:10.1016/j.ymthe.2020.12.004)
Supplement: Document S1. Tables S1–S3 and Figures S1–S6 [file mmc1.pdf]

YMTHE, Volume *29*

Supplemental Information

YAP Circular RNA, circYap, Attenuates Cardiac Fibrosis via Binding with Tropomyosin-4 and Gamma-Actin Decreasing Actin Polymerization

Nan Wu, Jindong Xu, William W. Du, Xiangmin Li, Faryal Mehwish Awan, Feiya Li, Sema Misir, Esra Eshaghi, Juanjuan Lyu, Le Zhou, Kaixuan Zeng, Aisha Adil, Sheng Wang, and Burton B. Yang

Table S1. General information of patients

Parameters		Values
donation hearts(n)		21
Ages (Mean±SD)		36.87±15.17
Gender		
	Male	15
	Female	6
Etiology		
	trauma	11
	other non-heart disease	10
Cardiac hypertrophy (n)		104
Ages (Mean±SD)		23.21±24.88
Gender		
	Male	63
	Female	41
Etiology		
	Heart failure	25
	Aortic stenosis	4
	Mitral stenosis	3
	Hypertrophic cardiomyopathy	8
	Tetralogy of Fallot	64

Table S2. Primer information.

Name		sequence
circYap junction	Forward:	5’-GCAAGAACTGCTTCGGCAGGTCCT-3’
	Reverse:	5’-GTTTATATAGTAAATTTCTCCATC-3’
circYap non-junction	Forward:	5'-GCAGCAACTGCAGATGGAGAAGGAG-3'
	Reverse:	5'-GGGTCTAGCCAAGAGGTGGTCTTG-3'
circYap linear precursor	Forward:	5'-TCTTCCTGATGGATGGGAAC-3'
	Reverse:	5'-GGCTGTTTCACTGGAGCACT-3'
Yap mRNA	Forward:	5’-CCGTGCCCATGAGGCTCCGGAAGC-3’
	Reverse:	5’-GGGTGTAGCTGCTGGGCCAGAGAC-3’
U6	Forward:	5'-GTGCTCGCTTCGGCAGCACATA-3'
	Reverse:	5'-TGGAACGCTTCACGAATTTGCG-3'
Collagen-I	Forward:	5'-GAATGGAGATGATGGGGAAGCTGG-3'
	Reverse:	5’-CATCTCCTTTGGCACCATCCAAAC-3'
Collagen-III	Forward:	5’-GTAAAGAAGTCTCTGAAGCTGATG-3'
	Reverse:	5’-GCGATATCTATGATGGGTAGTCTC-3'
TGF-β1	Forward:	5'-GAGCCTGAGGCCGACTACTA-3'
	Reverse:	5'-GGGTTCAGGTACCGCTTCTC-3'
CTGF	Forward:	5'-CAAGGGCCTCTTCTGTGACT-3'
	Reverse:	5'-ACGTGCACTGGTACTTGCAG-3'
NGF	Forward:	5'-AGGGAGCAGCTTTCTATCCTG-3'
	Reverse:	5'-GGC AGT GTC AAG GGA ATG C-3'
TNF-α	Forward:	5'-CCCAGGGACCTCTCTCTAATCA-3’
	Reverse:	5'-AGCTGCCCCTCAGCTTGAG-3’
TPM4	Forward:	5'-AAGTGTTGGGCAACAAAAGG-3'
	Reverse:	5'-CTTGGCAAAGTCAAGCTTCC-3'
ACTG	Forward:	5'-ATGTTGCCCTGGATTTTGAG-3'
	Reverse:	5'-AGGAAGGAAGGCTGGAAGAG-3'

Table S3. Mass spectrophotometry showing interaction of circYAP with proteins

Accession No.	Alternate ID	mol wt	vector	circYap	
O75367 (+2)	H2AFY	40 kDa	8.7988	5.0694	
P08238	HSP90AB1	83 kDa	14.665	5.0694	
P0CG39	POTEJ	117 kDa	69.413	0	
P11940 (+1)	PABPC1	71 kDa	14.665	4.0555	
P14625	HSP90B1	92 kDa	5.8659	2.0278	
P16989 (+2)	YBX3	40 kDa	5.8659	2.0278	
P24534	EEF1B2	25 kDa	2.9329	1.0139	
P27348	YWHAQ	28 kDa	2.9329	0	
P27482	CALML3	17 kDa	0	5.0694	←
P29692 (+1)	EEF1D	31 kDa	2.9329	0	
P31946 (+1)	YWHAB	28 kDa	5.8659	0	
P38159 (+1)	RBMX	42 kDa	5.8659	6.0833	
P47756	CAPZB	31 kDa	0	18.25	←
P51991 (+1)	HNRNPA3	40 kDa	14.665	6.0833	
P62258	YWHAE	29 kDa	3.9106	0	
P62807	HIST1H2BC	14 kDa	0	97.332	←
P62995	TRA2B	34 kDa	0	2.0278	←
P63104	YWHAZ	28 kDa	6.8435	3.0416	
P63261	ACTG1	42 kDa	0	191.62	←
P67936	TPM4	29 kDa	0	128.76	←
P68363 (+1)	TUBA1B	50 kDa	9.7765	4.0555	
P68366 (+1)	TUBA4A	50 kDa	6.8435	0	
P68371	TUBB4B	50 kDa	16.62	6.0833	
P68431	HIST1H3A	15 kDa	24.441	0	
P84243	H3F3A	15 kDa	0	30.416	←
Q04917	YWHAH	28 kDa	4.8882	0	
Q13595 (+2)	TRA2A	33 kDa	2.9329	1.0139	
Q13885	TUBB2A	50 kDa	12.709	7.0971	
Q15233 (+1)	NONO	54 kDa	0	6.0833	←
Q32MZ4-2 (+1)	LRRFIP1	86 kDa	0	4.0555	←
Q7Z794	KRT77	62 kDa	0	2.0278	←
Q8WXF1 (+1)	PSPC1	59 kDa	4.8882	2.0278	
Q96E39	RBMXL1	42 kDa	0	4.0555	←
Q9H361	PABPC3	70 kDa	7.8212	0	
Q9NYF8 (+2)	BCLAF1	106 kDa	0	2.0278	←
Q9P0M6	H2AFY2	40 kDa	6.8435	3.0416	

Arrows show proteins potentially interacting with circYap.

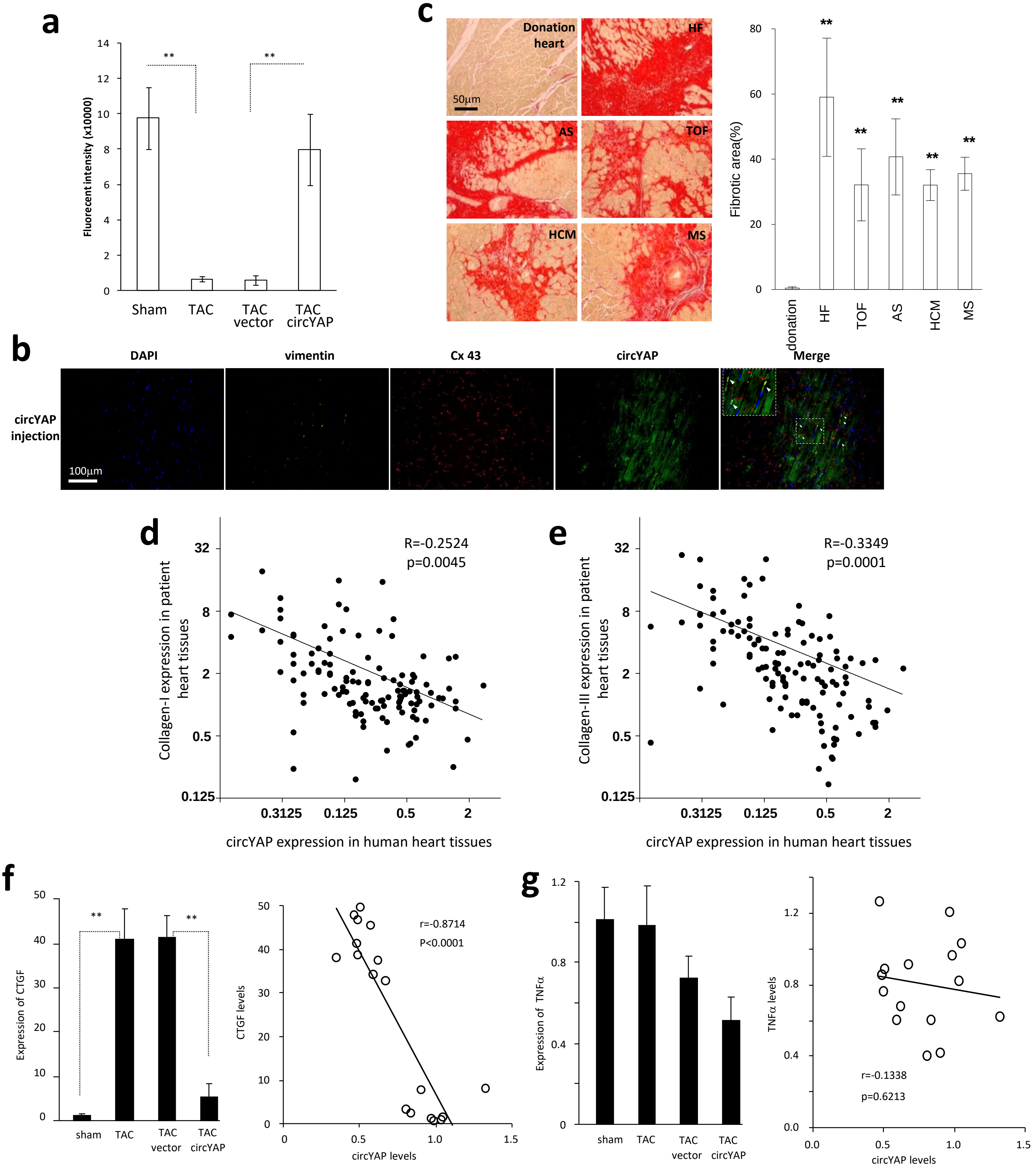

Fig S1. The circYap and fibrosis in heart.

(a) Quantitation of fluorescence in situ hybridization staining. n=4. **p<0.01.

(b) Co-localization of circYap with cardiac fibroblasts (marked with vimentin) and cardiomyocytes (marked with connexin) in mouse heart with TAC and circYap plasmid injection. Blue: DAPI; Yellow: Vimentin; Red, Cx43; Green circYap.

(c) Representative images of sirius red staining in human heart with or without cardiac hypertrophy. n=3.

(d-e) Correlation of circYap expression with collagen-I and collagen-II levels in human heart tissues. n=125

(f) Left: Expression of CTGF in mouse heart tissues of sham, TAC, TAC+vector and TAC+circYap mice. n=5. **p<0.01. Right: Correlation between CTGF and circYap levels n=18.

(g) Left: Expression of TNFα in mouse heart tissues of sham, TAC, TAC+vector and TAC+circYap mice. n=5. **p<0.01. Right: Correlation between TNFα and circYap levels n=16.

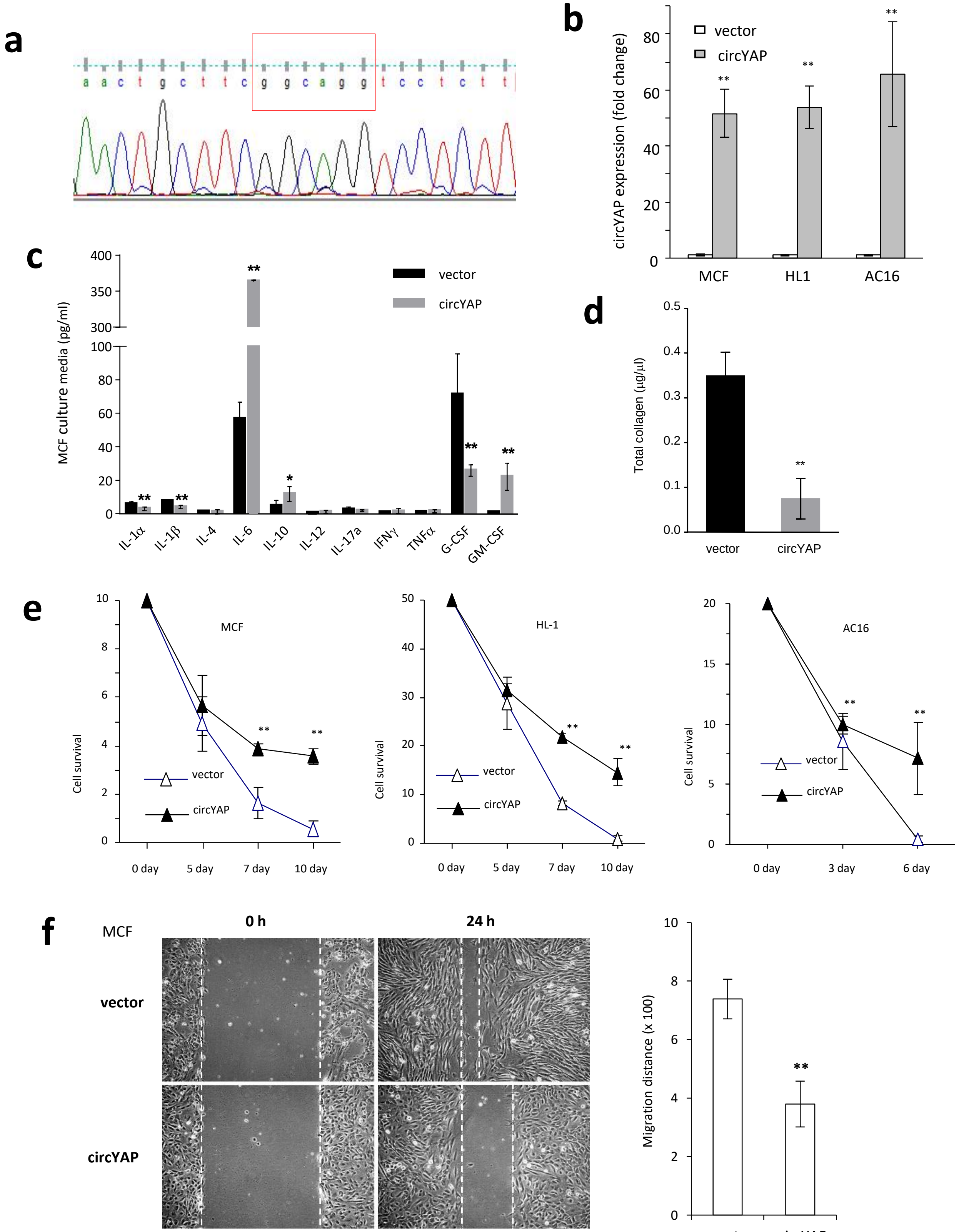

Fig S2. The role of circYap in heart cell function.

(a) Sanger sequence of the back-splice junction of ectopic human circYap.

(b) Ectopic expression of circYap in mouse cardiac fibroblasts (MCF), cardiomyocytes (HL-1) and human cardiomyocytes (AC16). n=4. **p<0.01.

(c) Cytokines that MCF secreted in cell culture media after TGF-β (1ng/ml) treated for 24h were detected using Qiagen multi-Analyte ELISArray Kit. n=6. **p<0.01, *p<0.05.

(d) Total collagen levels in cell culture media after TGF-β (1ng/ml) treated for 24h were detected using Abcam total collagen assay kit. n=5. **p<0.01.

(d) Overexpression of circYap constructs in MCF, HL-1 and AC16 cells increased cell survival. n=3. **p<0.01

(b) Effect of circYap on MCF migration. n=4. **p<0.01.

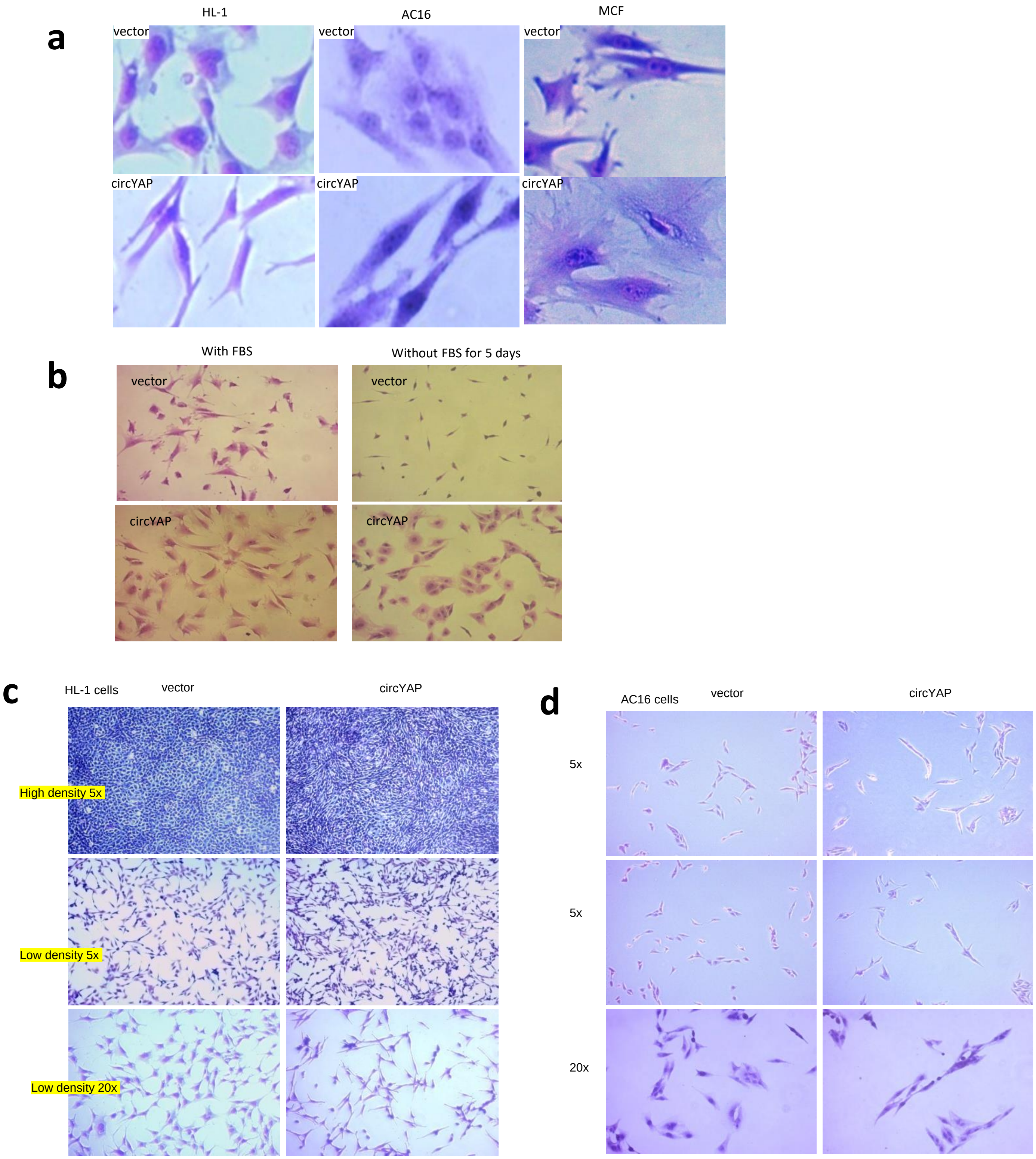

Fig S3. The role of circYap in change of cell morphology.

(a) Overexpression of circYap construct decreased cell spreading in HL-1 and AC16 cells, but increased cell spreading in MCF cells.

(b-d) Morphology changes after overexpression of circYap in HL-1, AC16 and MCF cells (large view).

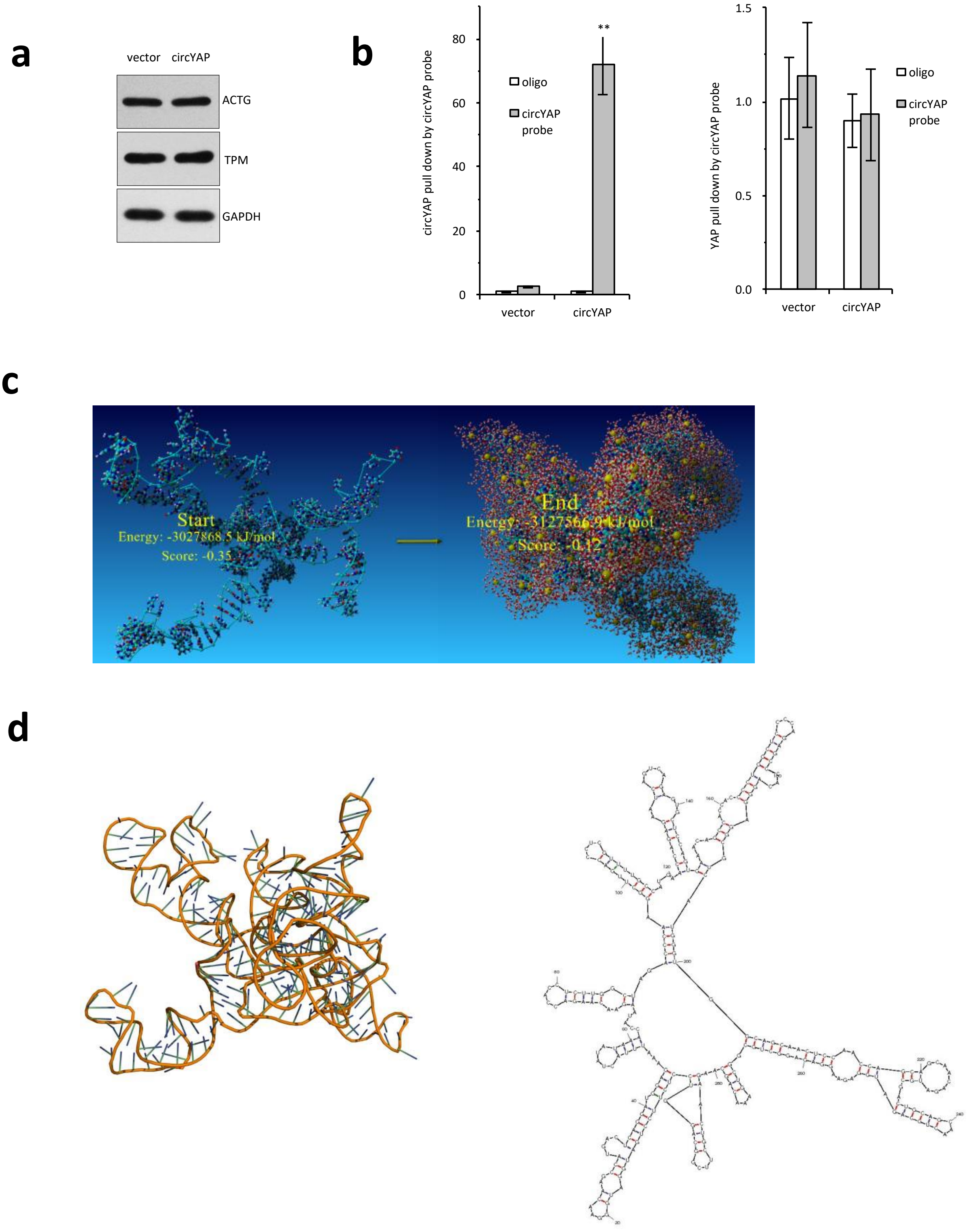

Fig S4. Two dimensional structure of circYap.
(a) Overexpression of circYap did not change levels of ACTG and TPM4.
(b) circYap probe could pull down circYap (left) but had no effect on pulling down linear YAP mRNA.
(c) YASARA representation of energy minimized 3D circ-YAP1 RNA.
(d) Two- and three-dimensional structures of circYap sequence.

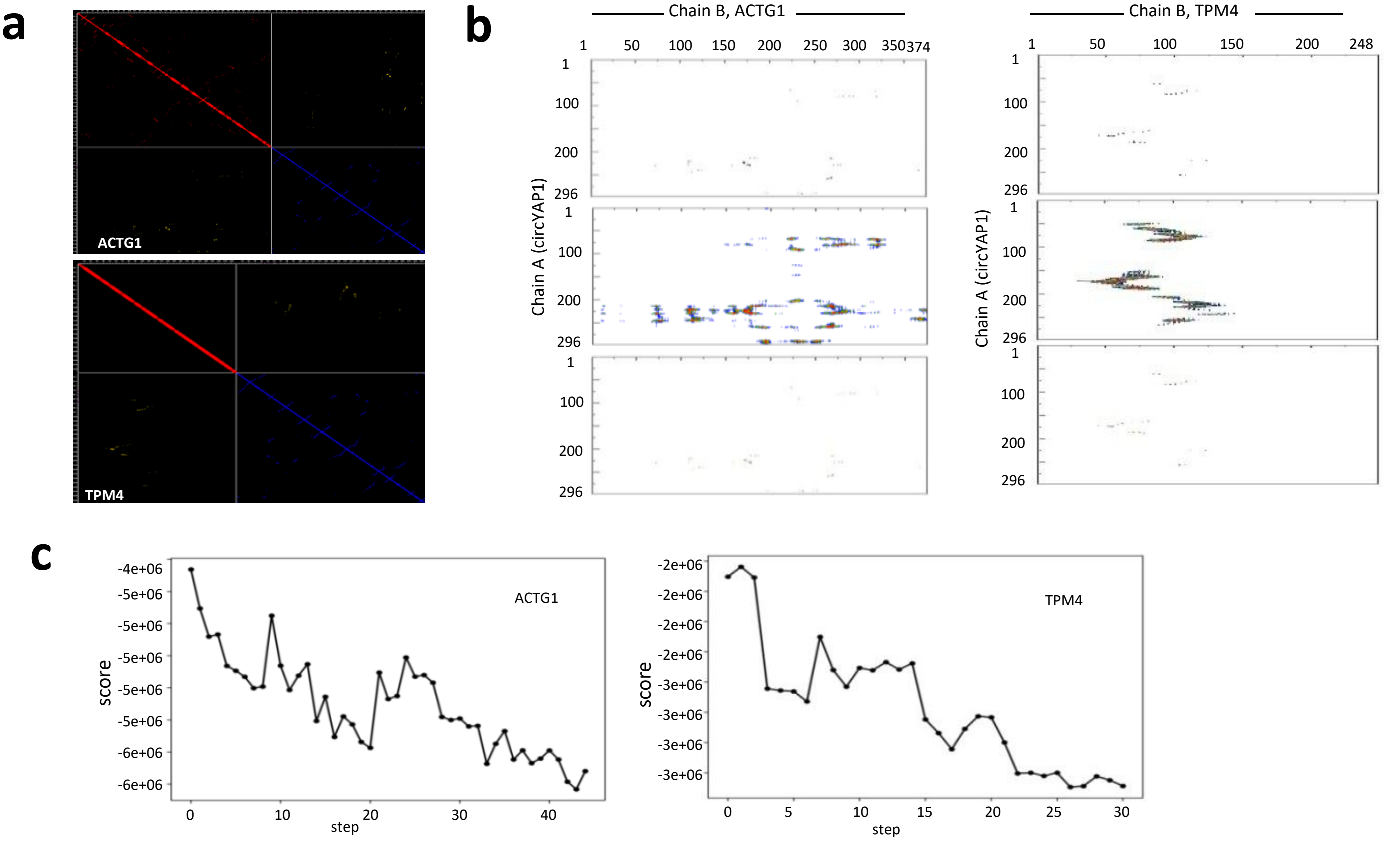

d

His-tagged ACTG construct with circYAP binding sites mutation
gccgccaccatggaagaagagatcgccgcgctggtcattgacaatggctccggcatgtgcaaagctggttttgctggggacgacgctccccgagccgtgtttccttccatcgtcgggcgccccagacaccagggcgtcatggtgggcatgggccagaaggactc
ctacgtgggcgacgaggcccagagcaagcgtggcatcctgaccctgaagtaccccattgagcatggcatcgtcaccaactgggacgacatggagaagatctggcaccacaccttctacaacgagctgcgcgtggccccggaggagcacccagtgctgctga
ccgaggcccccggcggaggcggaggcaacagagagaagatgactcagattatgtttgagaccttcaacaccccggccatgtacgtggccatccaggccgtgctgtccctctacgcctctgggcgcaccactggcattgtcatggactctggagacggggtcac
ccacacggtgcccatctacgagggctacgccggcggcggagccggcggtggtggtggactggctggccgggacctgaccgactacctcatgaagatcctcactgagcgaggctacagcttcaccaccacggccgagcgggaaatcgtgcgcgacatcaag
gagaagctgtgctacgtcgccctggacttcgagcaggagatggccaccgccgcatcctcctcttctctggagaagagctacgagctgcccgatggccaggtcatcaccattggcaatgagcggttccggtgtccggaggcgctgttccagcctggcggtctgggtg
gaggaggtggcggcatccacggaggcaccttcggaggtatcggtaagtgtgacgtggacatccgcaaagacctgtacgccaacacggtgctgtcgggcggcaccaccatgtacccgggcattgccgacaggatgcagaaggagatcaccgccctggcgcc
cagcaccatgaagatcaagatcatcgcacccccagagcgcaagtactcggtgtggatcggtggctccatcctggcctcactgtccaccttccagcagatgtggattagcaagcaggagtacgacgagtcgggcccctccatcgtccaccgcaaatgcttccacc
atcaccatcaccatcactaa
His-tagged TPM4 construct with circYAP binding sites mutation
gccgccaccatggccggcctcaactccctggaggcggtgaaacgcaagatccaggccctgcagcagcaggcggacgaggcggaagaccgcgcgcagggcctgcagcgggagctggacggcgagcgcgagcggcgcgagaaagctgaaggtgatg
tggccggcggtggaggaggcggtcagctcggtgaggagggtggtgacagggctggagaacgaggtggcacggccggtggaaagggtggagaggcagaaaaagctgcagatgagagtgagagaggaatgaaggtgatagaaaaccgggccatgaa
ggatgaggagaagatggagattcaggagatgcagctcaaagaggccaagcacattgcggaagaggctgaccgcaaatacgaggaggtagctcgtaagctggtcatcctggagggtgagctggagagggcagaggagcgtgcggaggtgtctgaactaa
aatgtggtgacctggaagaagaactcaagaatgttactaacaatctgaaatctctggaggctgcatctgaaaagtattctgaaaaggaggacaaatatgaagaagaaattaaacttctgtctgacaaactgaaagaggctgagacccgtgctgaatttgcagag
agaacggttgcaaaactggaaaagacaattgatgacctggaagagaaacttgcccaggccaaagaagagaacgtgggcttacatcagacactggatcagacactaaacgaacttaactgtatacaccatcaccatcaccatcactaa

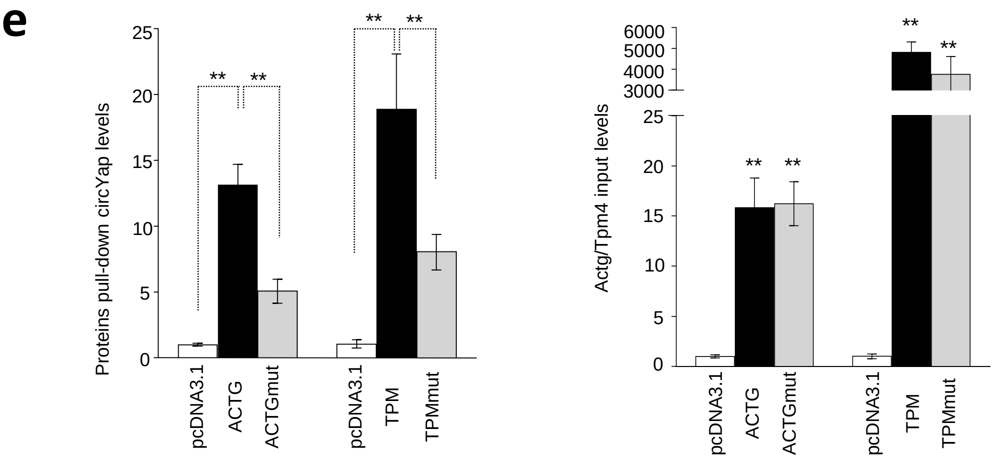

Fig S5. Computational analysis of circYap interacting with ACTG and TPM4.

(a) The contact map in the binding residues between circYap and ACTG or TPM4.

(b) The residue-level resolution contact maps in the binding residues between circYap and ACTG or TPM4.

(c) Refinement of the best docked circYap-ACTG model and circYap-TPM4 model showing MC score vs. steps of simulation.

(d) Plasmids that could be translated to His-tagged ACTG and TPM4 protein with or without circYap binding sites mutation (ACTGmut and TPM4mut) were transfected to circYap-overexpressed MCF cells. Ni-NTA beads were used to precipitate His-tagged ACTG and TPM4 and their bound circYap. n=6. **p<0.01.

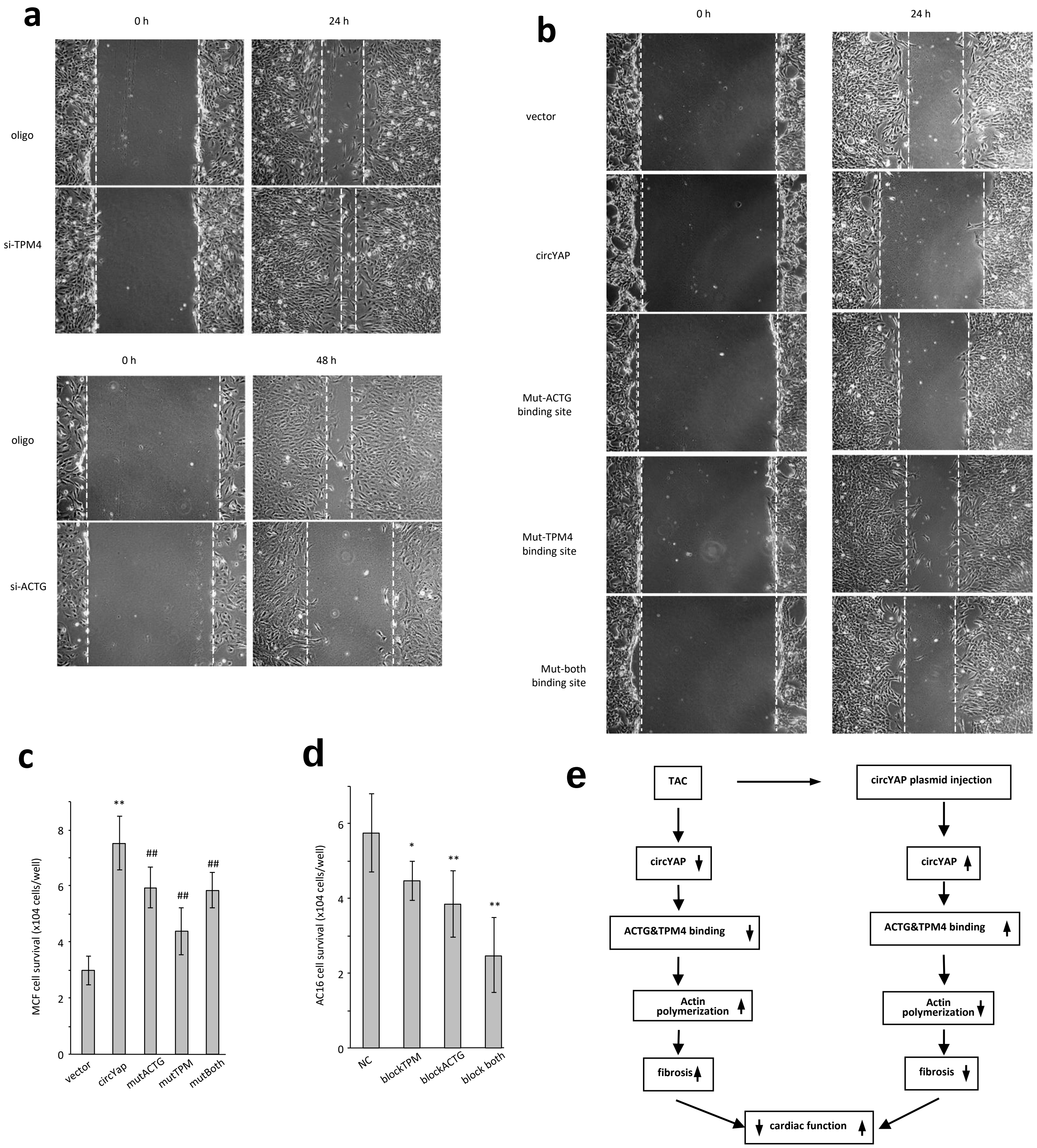

Fig S6. The role of circYap in cell migration

(a) Silencing TPM4 increased cell migration while silencing ACTG decreased cell migration.

(b) Overexpression of circYap decelerated cell migration while mutation of the binding sites in circYap abolished such effect.

(c) The survival rates upon serum deprivation in vector control, circYap, and plasmids containing mutations in the binding sites with ACTG and TPM4. n=6. **$p<0.01$ vs vector, $^{\#\#}p<0.01$ vs circYap

(d) The survival rates of AC16 cells transfected with the blocking oligos upon serum deprivation. n=6. **$p<0.01$ vs vector, $^{\#\#}p<0.01$ vs circYap.

(e) Diagram showing the mechanisms of circYap effects on heart fibrosis.
