# Supplementary material for: YAP Circular RNA, circYap, Attenuates Cardiac Fibrosis via Binding with Tropomyosin-4 and Gamma-Actin Decreasing Actin Polymerization
Source: Mol Ther. 2020 Dec 3;29(3):1138–50. doi: 10.1016/j.ymthe.2020.12.004 (PMC7934790; doi:10.1016/j.ymthe.2020.12.004)
Supplement: Document S2. Article and Supplemental Information [file mmc2.pdf]

Molecular Therapy
Original Article

YAP Circular RNA, circYap, Attenuates Cardiac Fibrosis via Binding with Tropomyosin-4 and Gamma-Actin Decreasing Actin Polymerization

Nan Wu,[1] Jindong Xu,[1,2] William W. Du,[1] Xiangmin Li,[1] Faryal Mehwish Awan,[3] Feiya Li,[1,4] Sema Misir,[1] Esra Eshaghi,[1] Juanjuan Lyu,[1] Le Zhou,[1] Kaixuan Zeng,[1] Aisha Adil,[1,4] Sheng Wang,[2] and Burton B. Yang[1,4]

[1]Sunnybrook Research Institute, Toronto, ON, Canada; [2]Department of Anesthesiology, Guangdong Provincial People's Hospital, Guangdong Academy of Medical Sciences, Guangdong Cardiovascular Institute, Guangzhou, Guangdong Province, China; [3]Department of Medical Lab Technology, The University of Haripur, Haripur, Pakistan; [4]Department of Laboratory Medicine and Pathobiology, University of Toronto, Toronto, ON M4N 3M5, Canada

Cardiac fibrosis is a common pathological feature of cardiac hypertrophy. This study was designed to investigate a novel function of Yes-associated protein (YAP) circular RNA, circYap, in modulating cardiac fibrosis and the underlying mechanisms. By circular RNA sequencing, we found that three out of fifteen reported circYap isoforms were expressed in nine human heart tissues, with the isoform hsa_circ_0002320 being the highest. The levels of this isoform in the hearts of patients with cardiac hypertrophy were found to be significantly decreased. In the pressure overload mouse model, the levels of circYap were reduced in mouse hearts with transverse aortic constriction (TAC). Upon circYap plasmid injection, the cardiac fibrosis was attenuated, and the heart function was improved along with the elevation of cardiac circYap levels in TAC mice. Tropomyosin-4 (TMP4) and gamma-actin (ACTG) were identified to bind with circYap in cardiac cells and mouse heart tissues. Such bindings led to an increased TPM4 interaction with ACTG, resulting in the inhibition of actin polymerization and the following fibrosis. Collectively, our study uncovered a novel molecule that could regulate cardiac remodeling during cardiac fibrosis and implicated a new function of circular RNA. This process may be targeted for future cardio-therapy.

INTRODUCTION

Cardiac fibrosis is one of the most common contributions to cardiac dysfunction, due to pathological myocardial remodeling. This process is associated with excessive matrix (i.e., collagens) deposition and cardiac fibroblast activation, leading to a variety of cardiac remodeling and progressive cardiac dysfunction.[1] Upon cardiac fibrosis, the compliance of heart tissues is decreased, and the progression of heart failure is accelerated. Cardiac fibroblasts play central roles in maintaining physiological heart functions as well as pathogenic cardiac remodeling during myocardial infarction and heart failure.[2,3] Following acute cardiac injury, fibroblasts are activated. These activated fibroblasts are involved in the process of inflammation, fibrosis, and scar formation consequently. In the past prevailing theory, the activated form of fibroblasts, also known as myofibroblasts, was regarded as the key effector in the pathogenesis of cardiac fibrosis via secreting collagens and other extracellular matrix (ECM) molecules. However, recent studies revealed protective effects of myofibroblasts upon pressure overload (PO).[4] There appear to be some factors regulating myofibroblast activities associated with synthesis of fibrosis markers and cardiac remodeling. Understanding the mechanism is critical for the development of safe and effective treatment targeting cardiac fibrosis.

Yes-associated protein (YAP) is an essential effector in the Hippo signaling pathway.[5–8] YAP has been reported to mediate crucial pathways in heart development, heart regeneration, cardiac hypertrophy, and myocardial infarction.[9–12] Activation of cardiomyocyte YAP could improve cardiac function and survival after cardiac injury.[13] In addition to functioning on cardiomyocytes, the Hippo-YAP pathway has been found to affect other types of cells in the heart, including fibroblasts, vascular cells, and immune cells. The human YAP gene regulates a variety of cell activities and can generate many exons.[14,15] With alternative splicing, many isoforms can be produced. Back-splicing of these exons can potentially synthesize 15 YAP circular RNA isoforms.[16] It thus appears that more circular RNA isoforms than mRNA isoforms can be synthesized by the same gene. In our previous study, we found that one of the YAP circular isoforms (hsa_circ_0002320), which is generated from exons 5 and 6 of YAP pre-mRNA, played a crucial role in cell proliferation and survival.[17] Interestingly, our circular RNA sequencing showed that this isoform was the most abundant YAP circular RNA (circYap) expressed in human heart tissue.

Received 3 April 2020; accepted 29 November 2020;
https://doi.org/10.1016/j.ymthe.2020.12.004.

Correspondence: Burton B. Yang, Department of Laboratory Medicine and Pathobiology, University of Toronto, S-Wing Research Building, 2075 Bayview Avenue, Toronto, ON M4N 3M5, Canada.
E-mail: byang@sri.utoronto.ca
Correspondence: Sheng Wang, Department of Anesthesiology, Guangdong Provincial People's Hospital, Guangdong Academy of Medical Sciences, Guangdong Cardiovascular Institute, Guangzhou, Guangdong, China.
E-mail: shengwang_gz@163.com

 © 2020 The Author(s).
This is an open access article under the CC BY license (http://creativecommons.org/licenses/by/4.0/).

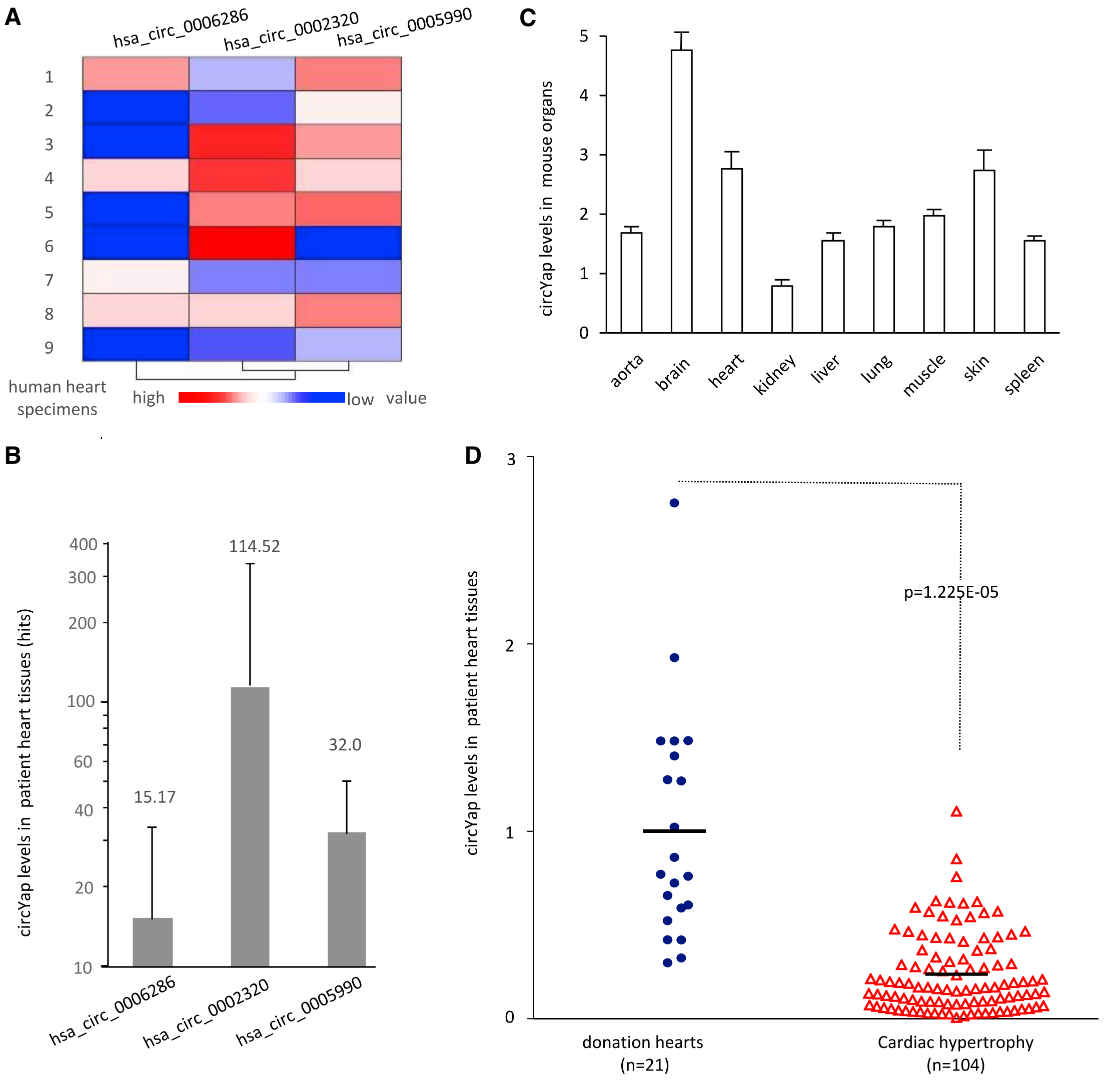

Figure 1. The Levels of circYap and Fibrosis Markers in Patient Heart Tissue

(A) Heatmap of nine human heart specimens expressing circular RNAs hsa_circ_0006286, hsa_circ_0002320, and hsa_circ_0005990, produced by YAP gene, performed by circular RNA sequencing. (B) Levels of the circular RNAs hsa_circ_0006286, hsa_circ_0002320, and hsa_circ_0005990. The number represented read count directly obtained by circular RNA sequencing. (C) Levels of circYap (hsa_circ_0002320) were measured by real-time PCR in different mouse organs. (D) Levels of circYap (hsa_circ_0002320) were measured by real-time PCR in 104 patient hearts with cardiac hypertrophy and 21 normal hearts from donations.

Recent studies have discovered the important roles of circular RNAs in regulating cardiovascular functions.[18–22] Considering the important function of YAP, the parental gene of circYap, in cardiovascular diseases and our previous study on circYap, we were inspired to further explore the roles of circYap in regulating cardiac functions. In particular, our sequencing results indicated that investigating the potential effects of circYap hsa_circ_0002320 in cardiac remodeling and fibrosis is imperative. Our present study is designed to discover the novel role of this circular RNA in the development of cardiac fibrosis and the possible underlying mechanisms.

RESULTS

Decreased Expression of circYap in Patients with Cardiac Diseases

Based on the RNA sequencing results in nine human heart specimens (W.W.D. et al., unpublished data), three out of fifteen reported circYap isoforms were found to be expressed in these human hearts (Figure 1A). Among these three isoforms, hsa_circ_0002320 was the highest expressed circYap isoform in human hearts (Figure 1B). Subsequently, we measured the levels of mouse equivalent (89% homology) in different mouse organs and found that all organs analyzed expressed this circYap isoform,

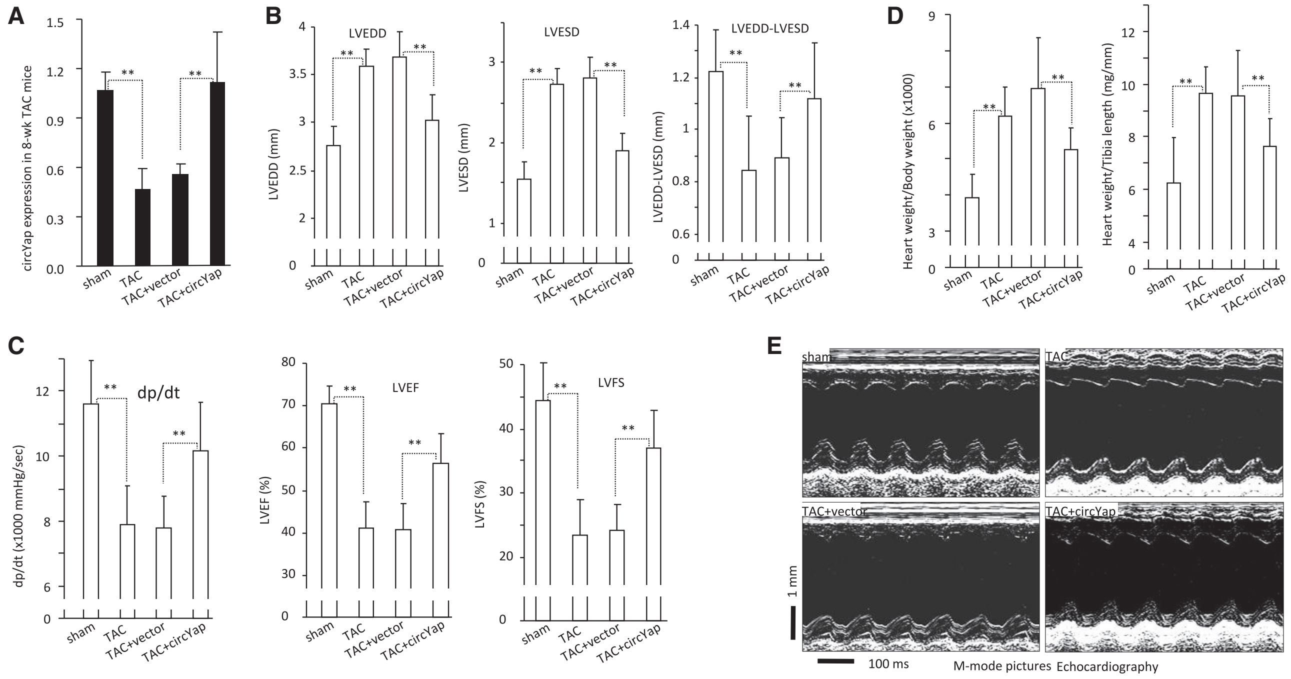

Figure 2. The Effects of circYap on Heart Function of Mice with Pressure Overload Induced by Transverse Aortic Constriction (TAC) Surgery

(A) Expression of circYap in mice with TAC and/or circYap plasmid injection for 8 weeks. (B) Echocardiography showed that TAC increased left ventricular end-systolic diameter (LVESD) and left ventricular end-diastolic diameter (LVEDD) compared to the sham mice. Such changes of heart function parameter could be improved by injection of circYap plasmid. n = 10. **p < 0.01. (C) TAC reduced contraction velocity (dp/dt), left ventricular ejection fraction (LVEF), and left ventricular fractional shortening (LVFS) compared to the sham mice, while ectopic delivery of circYap could prevent these effects of TAC. n = 10. **p < 0.01. (D) TAC increased heart weight to body weight or tibia length ratio that was eliminated by ectopic delivery of circYap. n = 10. **p < 0.01. (E) Representative photographs of echocardiography from sham, TAC, TAC + vector, and TAC + circYap plasmid injection mice.

with the heart being one of the organs expressing high levels (Figure 1C).

The YAP gene is known to play important role in maintaining heart functions.[10] We examined whether hsa_circ_0002320 expressed by the YAP gene had any clinical implication. The levels of circYap were measured in the hearts of 104 patients with cardiac hypertrophy, including 25 patients with heart failure, 4 patients with aortic stenosis, 3 patients with mitral stenosis, 8 patients with hypertrophic cardiomyopathy, and 64 patients with tetralogy of Fallot. As the controls, 21 heart samples from donations were also subjected to RNA isolation for real-time PCR. We found that the levels of circYap were significantly reduced in the hearts of patients with cardiac hypertrophy compared to those in the donated normal hearts (Figure 1D).

Ectopic circYap Improved Heart Functions in the TAC Mice

To test the role of circYap in maintaining cardiac functions, we generated PO in mice by transverse aortic constriction (TAC). We found the expression of circYap was significantly decreased in the heart 8 weeks after surgery, while ectopic delivery of circYap expression plasmid increased circYap levels (Figure 2A). In the following heart functional study, we detected a significant increase in left ventricular end-systolic diameter (LVESD) and the left ventricular end-diastolic diameter (LVEDD) in the TAC mice, which were diminished by ectopic delivery of circYap expression plasmids that were generated by us previously[17] (Figure 2B). We also detected a significant decrease in left ventricular ejection fraction (LVEF), left ventricular fractional shortening (LVFS), and contraction velocity (dp/dt) in the TAC hearts, and such reductions were prevented by the delivery of the circYap (Figure 2C). The heart weights were increased in the TAC mice but were maintained at normal levels in TAC mice with circYap plasmid delivery (Figure 2D). The M-mode pictures showed the typical increase in the left ventricular chamber of the TAC heart, and delivery of circYap reduced it to normal levels (Figure 2E).

Ectopic circYap Prevented Heart Fibrosis

Fluorescence *in situ* hybridization (FISH) confirmed successful delivery of circYap into mouse heart tissues (Figure 3A; Figure S1A). In addition, co-localization of circYap with cardiomyocytes and cardiac fibroblasts upon circYap plasmid delivery was observed by FISH and immunofluorescence (IF) staining (Figure S1B). H&E staining and Sirius red staining demonstrated that the hypertrophy and fibrosis in the TAC mouse hearts were prevented by the delivery of circYap plasmids (Figure 3B). Since cardiac hypertrophy and remodeling are associated with fibrosis, we stained the heart tissues with Masson trichrome and Sirius red to visualize the collagen deposition in the

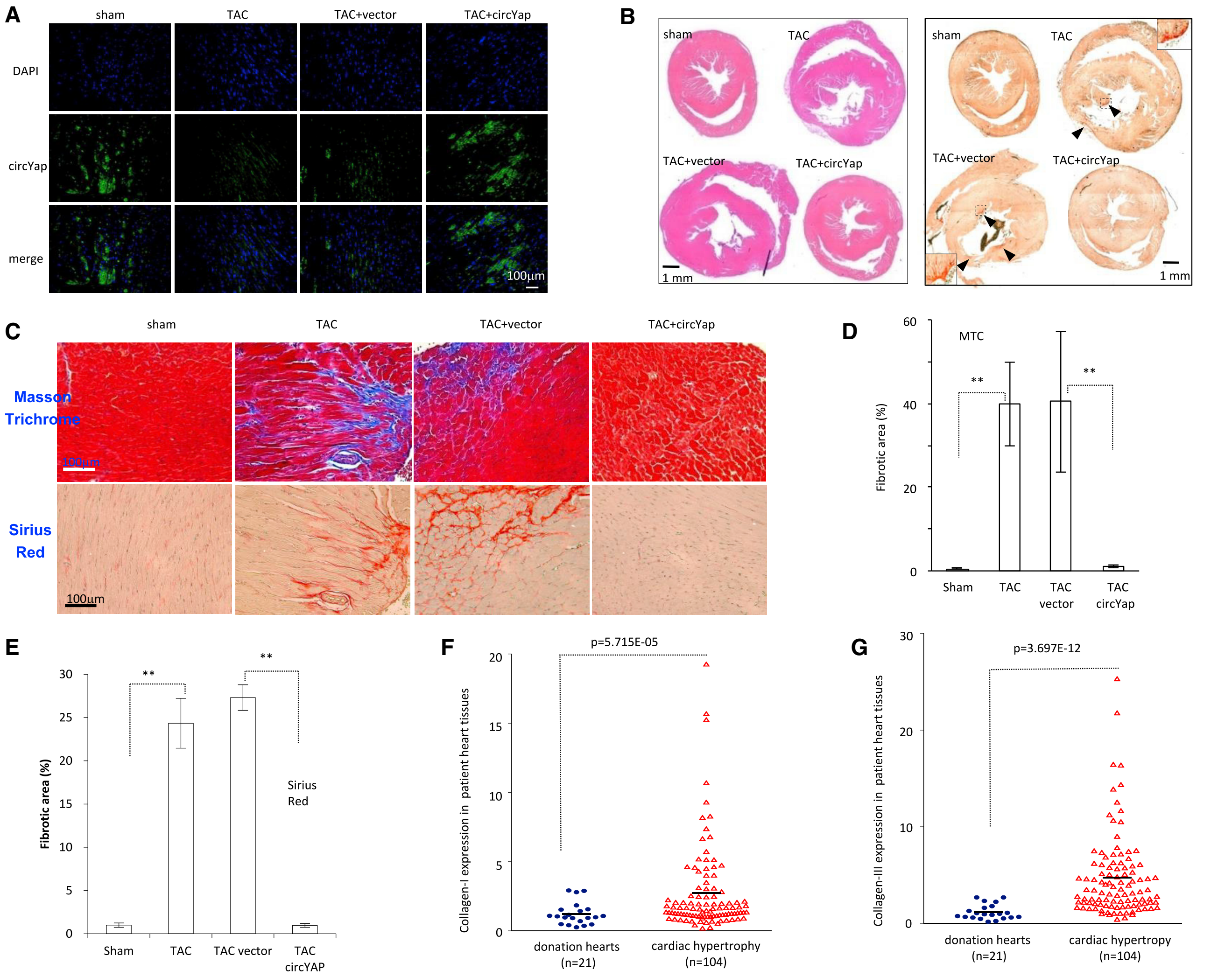

Figure 3. The Effects of circYap on Fibrosis in Pressure-Overload Hearts

(A) Representative photographs of fluorescence *in situ* hybridization (FISH) staining to confirm the successful circYap plasmid delivery into the mouse hearts. (B) Representative photographs of H&E-staining and Sirius red staining showed the heart sections of sham, TAC, TAC + vector, and TAC + circYap mice. (C) Representative photographs of Masson trichrome and Sirius red staining, showing that ectopic circYap delivery prevented the elevation of fibrosis in the TAC mouse hearts. (D and E) Quantitation of Masson trichrome (D) and Sirius red (E) staining. n = 3. $^{**}p < 0.01$. (F and G) Real-time PCR in patient hearts and normal heart donations showed that patients produced higher levels of collagen-I (F) and collagen-III (G) than the normal hearts.

heart tissues (Figure 3C). Quantitation analysis revealed that the collagen levels were significantly increased in the hearts of TAC mice, while circYap injection retained the collagen levels in TAC mice (Figures 3D and 3E). To examine the clinical relevance of these results, we measured levels of collagen-I and collagen-III in the patient heart specimens. The assays showed that the levels of collagen-I and collagen-III were significantly higher in the samples from the patients with cardiac hypertrophy compared to the hearts without cardiovascular disease (Figures 3F and 3G). In addition, the correlation of circYap and collagens in human heart tissue samples was analyzed. Moderate but significant negative correlations were observed between circYap and collagens (Figures S1D and S1E).

Furthermore, we analyzed collagen expression in mouse heart tissues by using real-time PCR. Collagen-I and collagen-III were significantly increased after TAC and decreased by circYap delivery (Figures 4A and 4B). The levels of both collagen-I (Figure 4A) and collagen-III (Figure 4B) were inversely correlated with circYap expression. We also measured the expression of other fibrosis markers, including transforming growth factor-β1 (TGF-β1) (Figure 4C), nerve growth factor (NGF) (Figure 4D), and connective tissue growth factor (CTGF) (Figure S1F). Expectedly, levels of all of these fibrosis markers increased significantly in the TAC mouse hearts. Ectopic delivery of circYap prevented such effects of TAC. The levels of these fibrosis markers showed inverse correlation with circYap levels. However, we did not find significant change

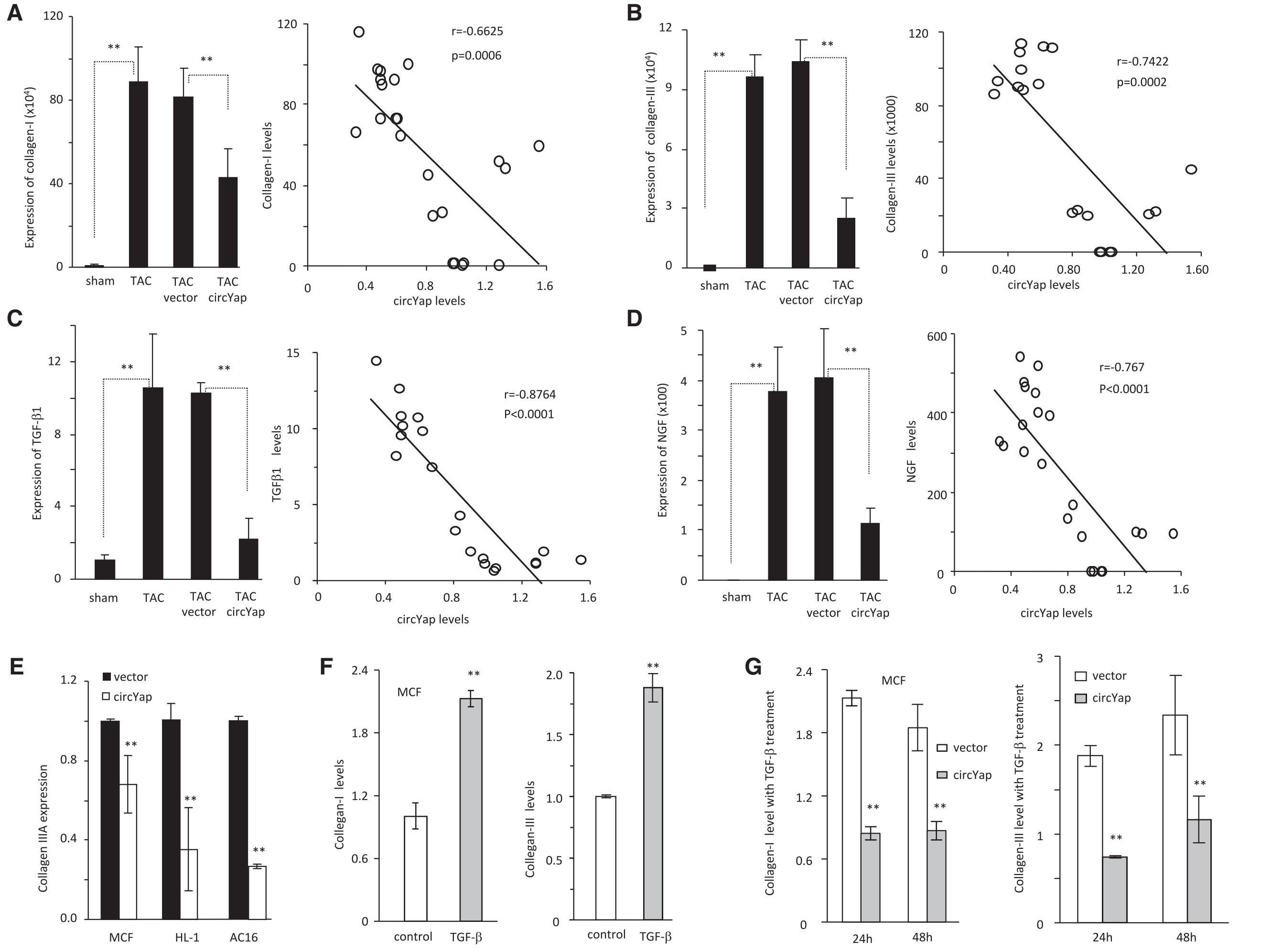

Figure 4. The Association of circYap with Fibrosis Markers

(A) Left: expression of collagen-I in mice heart tissues of sham, TAC, TAC + vector, and TAC + circYap mice. n = 5. **p < 0.01. Right: correlation between collagen-I and circYap levels. n = 23. (B) Left: expression of collagen-III in mice heart tissues of sham, TAC, TAC + vector, and TAC + circYap mice. n = 5. **p < 0.01. Right: correlation between collagen-I and circYap levels. n = 20. (C) Left: expression of TGF-β1 in mouse heart tissues of sham, TAC, TAC + vector, and TAC + circYap mice. n = 5. **p < 0.01. Right: correlation between TGF-β1 and circYap levels n = 20. (D) Left: expression of NGF in mouse heart tissues of sham, TAC, TAC + vector, and TAC + circYap mice. n = 5. **p < 0.01. Right: correlation between NGF and circYap levels. n = 22. (E) Decreased expression of collagen-III in MCF, HL-1, and AC16 cells transfected with circYap. n = 4. **p < 0.01. (F) Expression of collagen-I (left) and collagen-III (right) in MCF cells treated with TGF-β1. Treatment with TGF-β1 significantly increased collagen expression. n = 3, **p < 0.01. (G) MCF cells transfected with vector or circYap plasmid were treated with 1 ng/ml TGF-β1 for 24 or 48 h. Transfection with circYap decreased collagen-I (left) and collagen-III (right) levels. n = 3. **p < 0.01.

of tumor necrosis factor-α (TNF-α) levels, and therefore the levels of TNF-α were not correlated with circYap expression (Figure S1G). These results demonstrated that circYap was involved in maintaining heart function via inhibiting cardiac fibrosis during PO.

To examine how circYap affected heart functions, we ectopically expressed circYap in mouse cardiac fibroblasts (MCFs) and cardiomyocytes (HL-1 and AC16). After confirming the increased expression of circYap (Figures S2A and S2B), we measured mRNA expression of collagen-III and found the collagen-III mRNA expression was significantly downregulated in the cells with circYap overexpression (Figure 4E). Since fibroblasts play the most important roles in mediating the development of cardiac fibrosis, we treated MCFs with recombinant TGF-β1 to mimic the induction of fibrosis. The results showed that TGF-β1 treatment significantly boosted the expression of collagen-I and collagen-III (Figure 4F), while overexpression of circYap could successfully block the elevation of these fibrosis markers (Figure 4G). Overexpression of circYap also inhibited the total collagen levels in the culture media and altered the secretion of some cytokines upon TGF-β1 treatment (Figures S2C and S2D). In addition, overexpression of circYap significantly increased the survival rates of cardiac cells (Figure S2E) and decreased the migratory

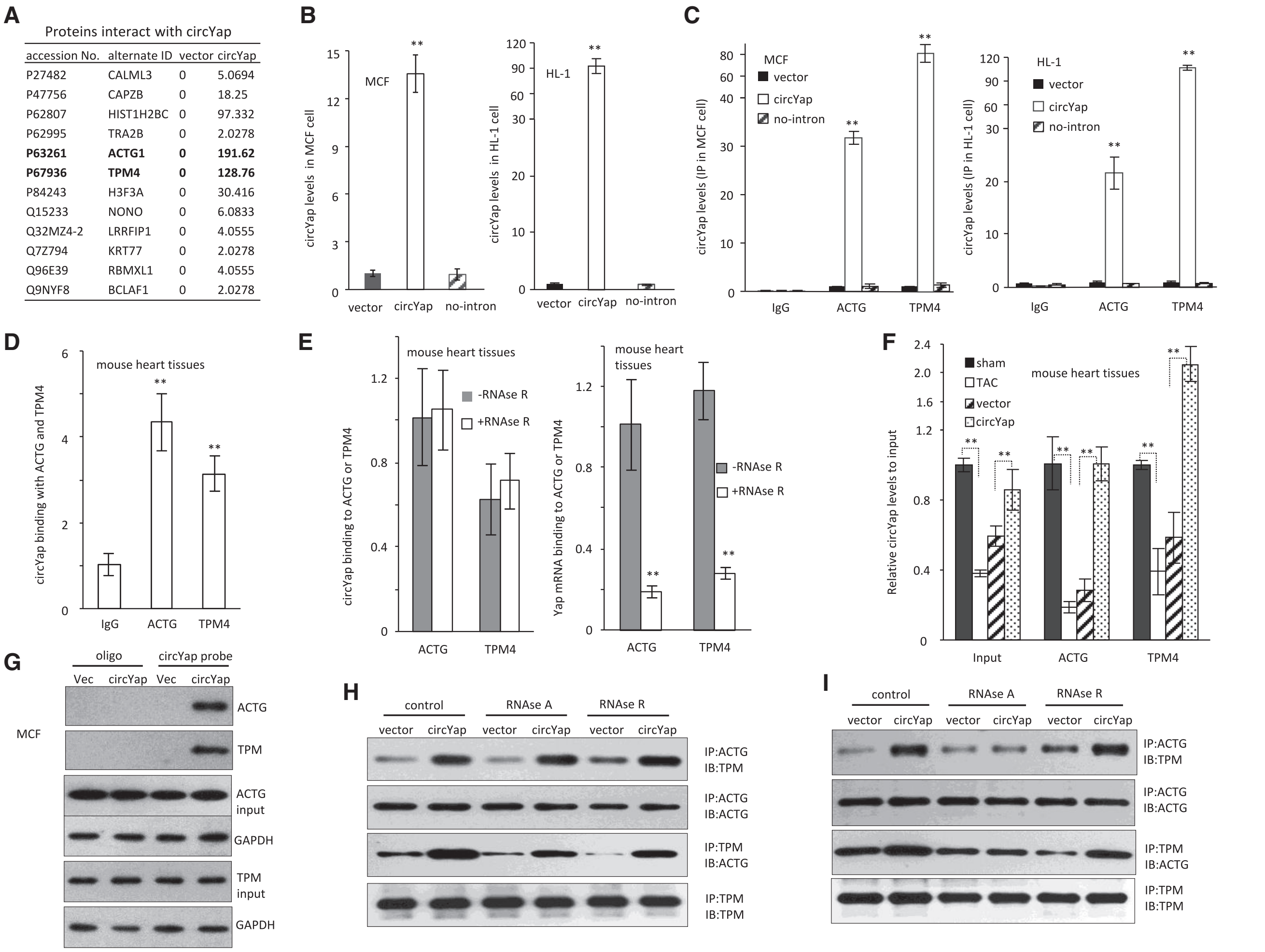

Figure 5. The Binding of circYap with ACTG and TPM4 Proteins

(A) Mass spectrometry assay showing the proteins pulled down by circYap probe. (B) Expression of circYap in MCF and HL-1 cells transfected with circYap or the plasmids lacking an intron for circularization (no-intron). n = 3. **p < 0.01. (C) The binding of circYap with ACTG and TPM4 in MCF and HL-1 cells with or without circYap and its linear precursor (no-intron) overexpression. n = 3. **p < 0.01. (D) Antibodies against ACTG and TPM4 could precipitate circYap in mouse heart tissues. n = 3. **p < 0.01. (E) The binding of circYap or YAP mRNA with TPM4 and ACTG in mouse heart tissues with or without RNase R treatment. n = 4. **p < 0.01. (F) The binding of circYap with ACTG and TPM4 in pressure-overload heart tissues with or without circYap plasmid injection. n = 4. **p < 0.01. (G) The circYap probe pulled down ACTG and TPM4 proteins in MCF cells. (H) The binding of ACTG and TPM4 proteins in MCF cells upon circYap overexpression with RNase A or RNase R treatment. RNase A and RNase R were added after immunoprecipitation. n = 3. (I) The binding of ACTG and TPM4 proteins in MCF cells upon circYap overexpression with RNase A or RNase R treatment. RNase A and RNase R were added before immunoprecipitation. n = 3.

ability of cardiac fibroblasts (Figure S2F). We also noticed the cell morphologies were obviously changed in the cells after stable transfection with circYap under both normal (with fetal bovine serum [FBS]) and stress (without FBS) condition. Upon circYap overexpression, the cardiac fibroblasts became flat and spread out, while the cardiomyocytes like HL-1 and AC16 cell were shrunken and elongated (Figure S3).

Interaction of circYap with TPM4 and ACTG

To uncover the molecules that mediated the function of circYap in cardiac fibrosis, we performed RNA pull-down assay by using the circYap probe in MCF and HL-1 cells. The precipitated proteins were identified by mass spectrometry analysis. Twelve proteins were identified to be pulled down by circYap probe, among which gamma-actin (ACTG) and tropomyosin-4 (TPM4) were ranked as the top two highest counts with no change in expression levels (Figure 5A; Table S3; Figure S4A). To confirm the mass spectrometry results and test the specificity of the interaction, we generated a circYap construct without the 5′ intron (no-intron), the essential component in back-splicing. Transfection with circYap increased circYap levels in both cell types significantly, but transfection with the no-intron construct had little effect (Figure 5B). MCF and HL-1 cells transfected

with circYap and the no-intron constructs were subjected to immunoprecipitation assays. Antibodies against ACTG and TPM4 proteins could precipitate circYap but not the product of the no-intron construct (Figure 5C). In mouse heart tissues, we also confirmed that antibodies against ACTG and TPM4 could precipitate endogenous circYap (Figure 5D).

Since we have reported that circYap has potential binding ability with YAP mRNA,[17] we used RNase R to treat the heart tissue lysate to exclude the possibility that circYap bound with these proteins via YAP mRNA. We found that after depletion of the potential binding of YAP mRNA with either ACTG or TPM4 by RNase R treatment, circYap could still bind to these two proteins (Figure 5E). This indicated that circYap directly bound to ACTG and TPM4 but not bound with these proteins via YAP mRNA. Moreover, we found that the binding of circYap with ACTG and TPM4 was significantly decreased in mouse heart tissues upon TAC but maintained at a higher level by restoration of circYap levels with plasmid injection (Figure 5F).

Due to the interaction of ACTG with TPM4 in a dynamic way under physiological conditions,[23] we examined the change of interaction of ACTG and TPM4 upon circYap overexpression. In the circYap-transfected cells, the circYap probe could pull down ACTG and TPM4 (Figure 5G; Figure S4B), which indicated that circYap enhanced the interaction of ACTG and TPM4.

To determine whether the interaction of ACTG and TPM4 were bridged by circYap or a complex of circYap-ACTG-TPM4 was formed, the reaction mixture was treated with RNase A or RNase R before or after the immunoprecipitation. We found that neither RNase A nor RNase R treatment after binding reaction could reduce the binding ability of ACTG with TPM4 (Figure 5H). RNase A treatment before the binding reaction showed decreased effect of circYap on the formation of the complex (Figure 5I). These results demonstrated that circYap directly bound to both ACTG and TPM4 and facilitated the interaction of these two proteins by forming a complex.

Identifying the Binding Sites of circYap with TPM4 and ACTG

By using a computational approach, the contact sites of circYap with ACTG and TPM4 were putatively mapped (Figure S4C). The best predicted secondary structure of circYap was analyzed for its thermodynamic properties. The structure of the ACTG protein used in the docking procedure was derived from Protein Data Bank (PDB) entry PDB: 5JLH. The molecular simulation result supported that circYap could perfectly dock ACTG, while the 2- and 3-dimensional sequence of the circYap showed different structures (Figure S4D). The docking analysis predicted a minimal binding region of circYap for ACTG, as shown in Figure 6A. Due to unavailable crystallographic data on TPM4, we used a homology modeling approach for the generation of the three-dimensional target structure. The molecular simulation also supported that circYap could perfectly dock TPM4, and this predicted a minimal binding region of circYap for TPM4, as shown in Figure 6B. Graphical representation of the three-dimensional structure of circYap-ACTG (PDB: 5JLH)-TPM4 complex was shown in Figure 6C and S5A–S5C. To confirm these binding activities, we performed site-directed mutagenesis to generate mutations of the binding sites in circYap or these two proteins, respectively. First, we transfected the cells with circYap or protein binding site mutated circYap constructs (circYapmuACTG and circYapmuTPM4). We confirmed that mutation of either or both protein binding sites for circYap significantly decreased the ACTG or TPM4 antibody precipitating circYap (Figure 6D). On the other hand, circYapmuACTG and circYapmuTPM4 could not pull down ACTG or TPM4 due to the mutation of their binding sites (Figure 6E). Moreover, transfection with circYapmuACTG and circYapmuTPM4 constructs remarkably reduced ACTG antibody precipitating TPM4 and vice versa (Figure 6F).

Second, we synthesized blocking oligos, which were reverse complement to the ACTG/TPM4 protein binding sites in circYap, to block the binding of circYap with ACTG or TPM4. It showed that ACTG and TPM4 were not able to precipitate circYap in the presence of the blocking oligos (Figure 6G). In accordance, transfection with the blocking oligos inhibited the capability of circYap to pull down ACTG and TPM4 proteins (Figure 6H). In addition, the blocking oligos also decreased antibody against ACTG precipitating TPM4 and vice versa (Figure 6I).

Last but not least, to further confirm the binding sites, we designed His-tagged ACTG or TPM4 with circYap binding sites mutation (ACTGmut and TPMmut) (Figure S5D). By using Hig-tag, the potential influence of site mutation on antibody or primer recognition of ACTG and TPM4 was avoided. We found that circYap could not pull down ACTG or TMP4 with the mutation of circYap binding sites (Figure 6J). In addition, ACTG or TPM4 with or without mutation were precipitated via His-tag by using Ni-NTA agarose beads. The mutated ACTG or TPM4 precipitated significantly less circYap compared to those without mutation (Figure S5E). These results confirmed that the binding of circYap and ACTG or TPM4 was abrogated by ACTGmut or TPMmut. In mouse heart tissues, the binding of ACTG and TMP4 were significantly suppressed in TAC mice that expressed decreased levels of circYap, while the interaction of ACTG and TPM4 in TAC mice with the ectopic delivery of circYap was maintained at similar levels to the sham mice (Figure 6K). These results indicated these protein binding sites were crucial for the binding of circYap with ACTG and TPM4, and circYap was involved in the interaction between ACTG and TPM4.

circYap Decreased Actin Polymerization

Actin polymerization plays important roles in regulating cell activities.[24] Tropomyosin is an essential regulator of actin polymerization.[25] Under physiological conditions, TPM proteins bind to actin to prevent the assembly of new actin filaments and actin polymerization. Our results showed that overexpression of circYap significantly inhibited the rate of actin polymerization (Figure 7A). In addition, we found that inhibition of ACTG by small interfering RNA (siRNA) could significantly reduce the migration rate of cardiac fibroblasts,

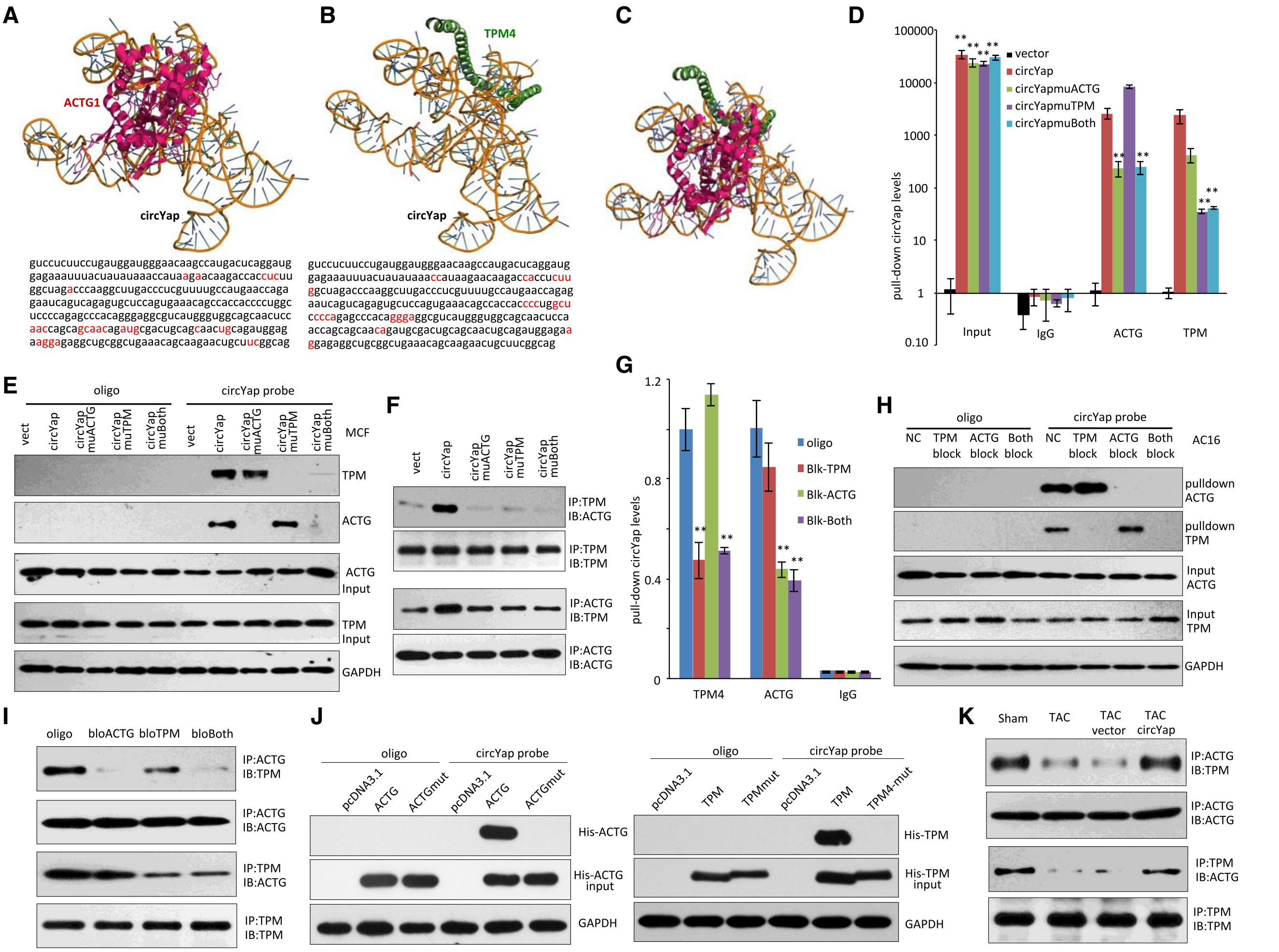

Figure 6. Identification of the Binding Sites of circYap with ACTG and TPM4

(A and B) The binding sites of circYap with ACTG protein (A) and TPM4 protein (B) were identified via computational approach. (C) Docking of the circYap-TMP4-ACTG complex. (D) RNA-immunoprecipitation by antibodies against ACTG and TPM4 was performed in MCFs transfected with circYap or mutant constructs. Mutations decreased the antibodies precipitating circYap. n = 4. $^{**}p < 0.01$. circYapmuACTG: ACTG binding site mutation in circYap; circYapmuTPM: TPM4 binding site mutation in circYap; circYapmuBoth: both ACTG and TPM4 binding sites mutation in circYap. (E) ACTG and TPM4 that bound with circYap were detected with respective antibodies after circYap was pulled down by its probe. ACTG and TPM4 proteins were pulled down by the circYap without binding sites mutation but not with binding sites mutation. circYapmuACTG, ACTG binding site mutation in circYap; circYapmuTPM, TPM4 binding site mutation in circYap; circYapmuBoth, both ACTG and TPM4 binding sites mutation in circYap. n = 3. (F) Antibody against ACTG could precipitate TPM4 and vice versa with circYap overexpression. Binding sites mutation of circYap decreased the interactions of ACTG with TPM4. n = 3. (G) RNA-immunoprecipitation of circYap by antibodies against ACTG and TPM4 was performed in AC16 cells transfected with blocking oligos that reverse complement to the ACTG or TPM4 binding sites. n = 4. $^{**}p < 0.01$. (H) ACTG and TPM4 were pulled down by the circYap in AC16 cells transfected with the blocking oligos. The blocking oligos inhibited circYap pulling down the ACTG and/or TPM4 proteins. n = 3. (I) Transfection with the blocking oligos inhibited ACTG precipitating TPM4 and vice versa. n = 3. (J) Plasmids that could be translated to His-tagged ACTG and TPM4 protein with or without circYap binding site mutation (ACTGmut and TPM4mut) were transfected to circYap-overexpressed MCF cells. The circYap could not pull down ACTG or TPM4 with the binding site mutation. n = 3. (K) In the heart tissue of TAC mice, decreased expression of circYap inhibited ACTG precipitating TPM4 and vice versa. Ectopic delivery of circYap plasmids increased the interaction of ACTG and TPM4. n = 3.

while silencing TPM4 by siRNA could remarkably accelerate the migration (Figure 7B; Figure S6A). This result confirmed the inhibitory effects of TPM4 on ACTG-mediated cell activities by polymerization. We further validated that interrupting the binding of circYap with ACTG and TPM4 by mutation on the binding sites significantly increased the motility of cardiac fibroblasts (Figure 7C; Figure S6B) and elevated the collagen-I and collagen-III levels (Figures 7D and 7E). In addition, blocking the binding of circYap to ACTG and TPM4 by either mutation, the binding sites or transfection with the blocking oligos decreased viability of MCF or AC16 cells (Figures S6C and S6D).

Finally, we tested whether circYap-mediated actin polymerization could occur *in vivo*. Heart lysates were prepared from sham and

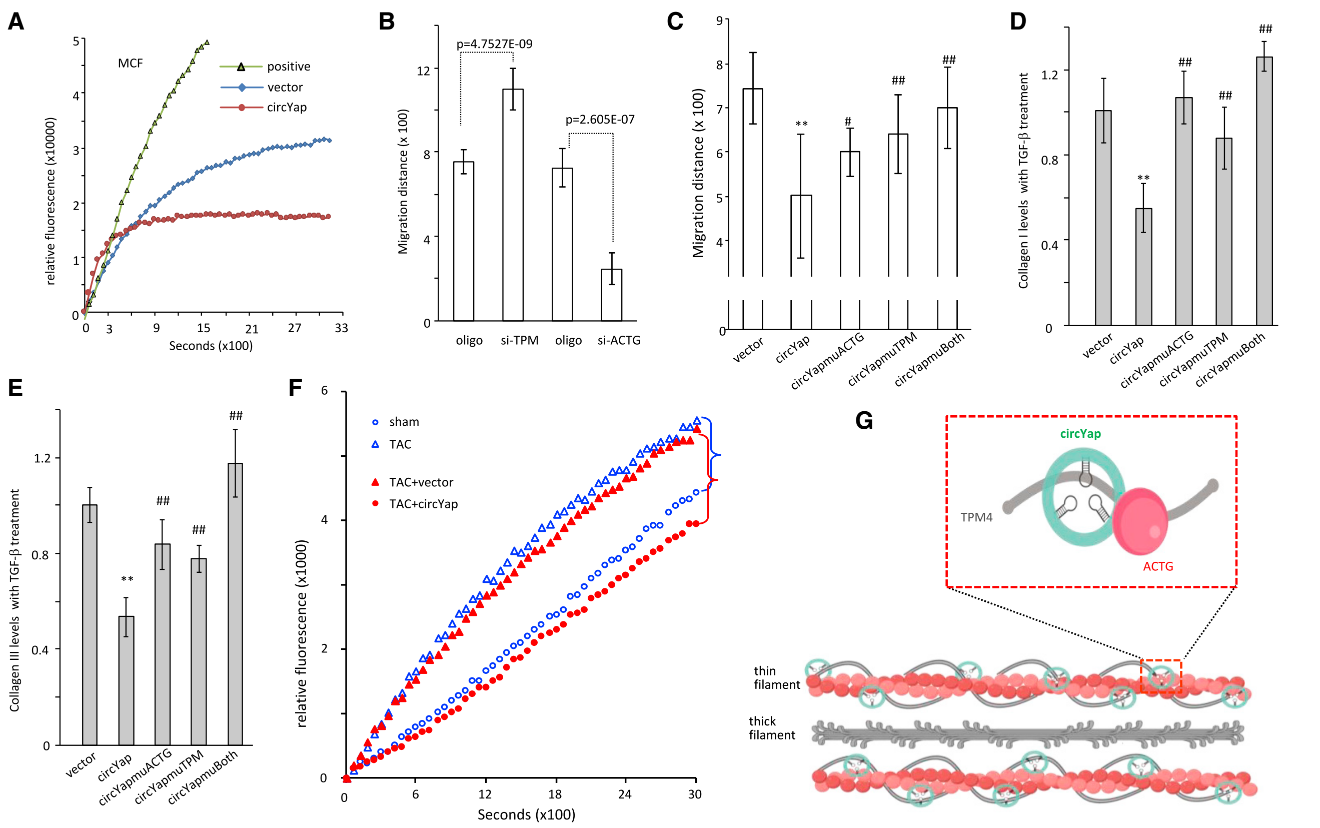

Figure 7. The Function Change of Heart Cells upon siRNA and Binding Sites Mutation

(A) Actin polymerization rates in MCF cells transfected with control vector or circYap plasmids. Expression of circYap decreased actin polymerization. (B) The migration rates of MCF cells transfected with TPM4 siRNA, ACTG siRNA, or control oligo were tested by wounding assays. Silencing TPM4 increased cell migration, while silencing ACTG decreased cell migration. n = 7. $^{**}p < 0.01$. (C) The migration rates of MCF cells transfected with vector control, circYap, and circYap constructs containing binding site mutations with ACTG and TPM4 (circYapmuACTG and circYapmuTPM). n = 9. $^{**}p < 0.01$ versus vector, $^{\#\#}p < 0.01$ versus circYap. (D and E) The levels of collagen-I and collagen-III in vector control, circYap, and circYap constructs containing binding site mutations with ACTG and TPM4 (circYapmuACTG and circYapmuTPM). n = 4. $^{**}p < 0.01$ versus vector, $^{\#\#}p < 0.01$ versus circYap. (F) In the heart tissues of TAC mice, reduction of circYap expression increased actin polymerization. Ectopic delivery of circYap decreased actin polymerization. (G) Diagram of circYap binding with ACTG and TPM4 forming complexes, playing roles in actin polymerization and tissue contraction.

TAC mice that showed decreased expression of circYap. An increase of actin polymerization was found in TAC mouse heart with the reduction of circYap expression. Ectopic delivery of circYap could inhibit the actin polymerization (Figure 7F). Therefore, we concluded that circYap facilitated the binding of TPM4 with ACTG, leading to the increased inhibitory effect of TPM4 on actin polymerization and the following anti-fibrotic effects (Figure 7G; Figure S6E).

DISCUSSION

Cardiac fibrosis is a worldwide health problem associated with nearly all etiologies of heart diseases. However, the molecular mechanisms underlying cardiac fibrosis remain unclear. Our study indicates that circYap is a critical regulator in binding to tropomyosin and actin, decreasing actin polymerization and cardiac fibrosis. Our study also suggests that we could target the process of actin polymerization to decrease cardiac fibrosis and improve heart functions by ectopic delivery of nanoparticle-conjugated circYap expression plasmids.

The promising potential of RNA-targeting therapeutics for human diseases has started to be unveiled in recent years. Circular RNAs have been reported to affect progression of heart disease and cancer.[26–30] As the most stable RNAs due to their special structure, circular RNAs are potential targets in RNA therapeutics.[31–33] The circYap is one of the circRNAs that we found to have protective effects in both cancer[17] and cardiovascular disease. Potentially, the human YAP gene can produce 15 isoforms of circular RNAs.[16] However, the expression of circYap could be tissue specific, since our circular RNA sequencing result showed that only three isoforms were detected in human heart tissues. Therefore, we selected the one with the highest level in our study. We found that the level of this circYap isoform was significantly decreased in the hearts of patients with cardiac hypertrophy. In addition, our results revealed that the expression of circYap was negatively correlated with fibrosis factors including collagen-I and -III, TGF-β, CTGF, and NGF in the heart of TAC-induced PO mouse model. In the present study, we successfully restored the circYap levels in the hearts of mice with TAC by delivery of circYap plasmids, which

were validated by real-time PCR and FISH. Notably, the impaired heart function and the cardiac fibrosis that resulted from PO were significantly improved after ectopic circYap plasmid delivery. These results provided a clue for the potential clinical application of circYap in RNA therapeutics. Regarding the cellular target of circYap in the heart, our present study showed that circYap affected the survival and cell morphology of both fibroblasts and cardiomyocytes in *in vitro* cell models. The fibroblasts are acknowledged to be the major effectors of cardiac fibrosis. It has been reported that fibroblasts can modulate cardiomyocyte survival via transduction of hypertrophic signal or secretion of pro-apoptotic, pro-inflammatory mediators and exosomes.[34] Our results from FISH in mouse heart tissue indicated circYap is expressed in both cardiomyocytes and cardiac fibroblasts. However, the function of circYap on myocytes and fibroblasts may be slightly different due to the different roles of these two types of cells in cardiac remodeling. The roles of circYap on specific cell types warranted further investigation in future studies.

In our previous study, we discovered that circYap inhibited YAP protein expression in breast cancer.[17] YAP protein expression was increased in tumor tissues/cells compared to the benign tissue/normal cells. Elevation of circYap could effectively reduce YAP protein expression to the level of control tissues or cells. In the current study, however, YAP mRNA and protein were found to be decreased in parallel to circYap in TAC mouse heart; therefore, circYap is unlikely to antagonize YAP expression itself. The mechanisms of circYap on the YAP signaling pathway in cancer cannot explain the beneficial effects of circYap on heart function. Therefore, other potential mechanisms that mediated the function of circYap in the PO model were investigated in the present study. Here, we demonstrated that circYap inhibited cardiac fibrosis via controlling the interaction of ACTG and TPM4 and the following actin polymerization. TPM plays a critical role in actin polymerization by stabilizing actin filaments. It has been reported that mutation of TPM could result in hypertrophic cardiomyopathy, dilated cardiomyopathy, or left ventricular noncompaction.[35] TPM also inhibited new actin filament assembly via restricting the ability of the Arp2/3 complex to nuclear actin polymerization.[36] In our study, we found that circYap could bind with both TPM4 and ACTG to form a complex and significantly enhanced the inhibitory effect of TPM4 on actin polymerization. By binding with circYap, the interaction of TPM and ACTG was significantly enhanced. Therefore, TPM played its critical role in actin filament assembly and thus suppressed actin polymerization. Upon TAC-induced PO, actin polymerization in the mouse heart tissue was accelerated, leading to the development of collagen deposition and fibrosis. Ectopic delivery of circYap in our study significantly decreased this process. It has been reported that enhancement of actin polymerization could activate the process of lung fibrosis.[37] Here, our study confirmed the link between actin polymerization and cardiac fibrosis and identified circYap as the potential inhibitor to prevent the development of cardiac fibrosis via deactivating actin polymerization.

Although our results showed that circYap could bind to ACTG and TPM4, we did not examine the sizes of the binding complexes. Since actin can form filaments and tropomyosin can bind to actin, there is a possibility that the complexes of circYap-ACTG-TPM4 have different sizes, which depends on the interaction of actin-actin and actin-tropomyosin. The sizes of the complexes may contribute to actin polymerization and cardiac fibrosis. Additional technologies with innovation are needed to uncover the details of the interaction in the future. Due to the potential formation of large complexes of these three molecules, it is also possible that some circYap-binding proteins detected by mass spectrophotometry may be under-representative. Whether these proteins are involved in circYap-regulating cardiac fibrosis warrants further investigation. Moreover, we demonstrated that ectopic circYap elevated the circYap levels in both cardiomyocytes and cardiac fibroblasts *in vivo* and altered the morphology of cardiomyocytes and cardiac fibroblasts *in vitro*. Since cardiac fibroblasts are acknowledged to play the major role in cardiac fibrosis,[38] we consider the protective effects of circYap on heart function and cardiac fibrosis here is mainly mediated by the inhibition of actin polymerization in cardiac fibroblasts. Our present study may offer a novel and promising approach to impede cardiac fibrosis and improve the heart function by targeting circRNA.

MATERIALS AND METHODS

Human Heart Specimens

Heart samples were obtained from 104 patients with cardiac hypertrophy resulting from heart failure, aortic stenosis, mitral stenosis, hypertrophic cardiomyopathy, or tetralogy of Fallot, and 21 organ donations without heart disease records (trauma or non-cardiac causes). The clinical characteristics and etiology of these patients are described in Table S1. The donation hearts from individuals who died from non-cardiac causes were collected 0.5–6 h after patient's death. At the time of surgery, the heart tissue was removed from the left ventricular free wall or right ventricular outflow tract and snap-frozen in liquid nitrogen for RNA or protein isolation. The study was carried out in accordance with the Ethics Code of the World Medical Association (Declaration of Helsinki). All patients selected in this study gave formal informed consent prior to enrollment.

Animal Models

The PO model was induced by modified TAC in mice as previous described.[39] A successful PO model was confirmed with both visual confirmation of differential carotid pulpability and measuring the carotid artery flow velocities by Doppler. Those mice with a right carotid (RC)/left carotid (LC) flow ratio >5 were included for further experiments. The sham mice underwent surgery at the same time points with the similar process of anesthesia and other operation except aortic banding. All animal experiments were performed in accordance with relevant guidelines and regulations approved by the Animal Care Committee of Sunnybrook Research Institute.

For circYap administration, 8-week-old C57BL6 mice were processed to TAC and the plasmids of control vector or circYap (50 μg each) were injected intraperitoneally twice per week for 8 weeks. The plasmids of circYap and vector control were generated as described in the following section of construct generation. The plasmids were

conjugated with polyethylene glycol (PEG) and AU nanoparticles before injection as previously described.[40] Groups of sham and TAC mice without injection served as controls. After 8 weeks of injection, mice were sacrificed following cardiac function assessment. Hearts and other organs were harvested. Part of the heart tissue was fixed with 10% buffered formalin and embedded in paraffin and then sectioned to 5 μm slides. H&E staining, Masson's trichrome staining, and Sirius red staining were performed as described.[18]

Mice were anesthetized with 2% isoflurane inhalation to undergo transthoracic echocardiography and invasive hemodynamic assessment. Transthoracic echocardiography was performed and analyzed in a blinded manner, using a Vevo 2100 high-resolution imaging system equipped with a 40-MHz transducer to measure LVEDD, LVESD, LVEF, LVFS, and dp/dt. A 1.4-Fr high-fidelity pressure catheter (SPR-671, Millar Instruments, Houston, TX, USA) was inserted into the LV via the right carotid artery to evaluate left ventricular pressure (LVSP) and dp/dt using PowerLab system (AD Instruments).

For Sirius red staining, mouse heart sections were stained with Weigert's hematoxylin for 8 min after being dewaxed and hydrated. Then, the slides were stained in 0.1% picrosirius red for another 1 h and washed in 0.1% acetic acid. Masson's trichrome staining was performed with Masson's trichrome staining kit from American Master Tech.

FISH

In FISH, Alexa 488 labeled DNA oligo probes against circYap were generated by fluorescence PCR labeling kit from Biolynx. The labeled probes were heated at 95°C for 5 min and chilled on ice immediately to prevent reannealing. A scramble sequence was used as a negative control. After dehydration and air drying, the fixed samples were pretreated with hybridization solution in 55°C for 0.5 h. The pre-hybridized slides were incubated with 50 nM fluorescence-labeled DNA oligo probes in hybridization buffer at 55°C for 1 h followed by serial washes with saline-sodium citrate (SSC) buffers. DAPI staining was done for another 20 min after washing with PBS before mounting.

RT-PCR and RNase Treatment

Total RNA was extracted from cells or tissues using a kit from Geneaid TriRNA isolation kit or TRIzol. 1 μg RNA was subjected to reverse transcription and quantitative PCR (qPCR) using iScript RT kits and SYBR green master mix (Bio-Rad). U6 or GAPDH were used as internal controls. The sequences of primers are listed in Table S2. RNase treatment was conducted as previously described.[41] In brief, 1 μg RNase R (Epicenter) or RNase A (QIAGEN) was added in the mixture either before or after immunoprecipitation and incubated at 37°C for 15 min.

RNA and Protein Interaction

For immunoprecipitation, 100 μL magnetic beads were washed in PBS-T (PBS + 0.1% Tween-20) and incubated with 1 μg primary antibody at room temperature for 10 min. The total protein lysates were collected, and the protein concentrations of different samples were equalized. Then, the protein lysate was incubated with antibody-containing beads for 1 h. The magnetic beads were washed 3 times with PBS-T and were resuspended in either TRIzol (for RNA extraction) or 2 × Laemmli buffer (0.125 M Tris-HCl, 4% SDS, 20% glycerol, 10% 2-mercaptoethanol, 0.004% bromphenol blue [pH 6.8] for protein isolation). The isolated RNAs were subjected to real-time PCR, while the isolated proteins were subjected to western blotting.

RNA pull-down assay was performed using an RNA probe as described.[42] In brief, the cells were lysed in coimmunoprecipitation (coIP) buffer and then incubated with 3 μg biotinylated DNA oligo probes against circYap at room temperature for 2 h. 50 μL Streptavidin C1 magnetic beads (Invitrogen) were added to each binding reaction and further incubated at room temperature for another 1 h. The beads were washed briefly with coIP buffer 5 times. The bound proteins in the pull-down material were analyzed by western blotting. The oligomers for RNA pull-down of human circYap 5′-tcaggaagaggacctgccgaagcagttcttgc were biotinylated using the biotin-11-dATP labeling kits.

Bioinformatics Prediction

To determine the possible interaction of circYap with ACTG1/TPM4, 20,000 models were generated using NPDock server,[43] a protein-RNA docking analysis tool. NPDock server combines GRAMM for global macromolecular docking, scoring with a statistical potential, and clustering, followed by refinement of best-scored docked complexes from the three biggest clusters. Distance-based and residue-level resolution contact maps of circYap-ACTG1 docked complex and circYap-TPM4 docked complex were determined using RNAmap2D[44] and COCOMAPS[45] tools. Contact distances were computed between Cα atoms of protein residues and O5′ atoms of RNA strands. Two residues are in contact when their O5′–Cα distance is less than 10 Å. A distance-based approach was used to identify the binding site residues/nucleotides for the protein-RNA complexes using a specific cutoff value. Two atoms (one in RNA and another in protein) are considered to be interacting with each other if the distance between them is <3.5 Å.

Actin Polymerization Assay

Actin polymerization was conducted with the Actin Polymerization Biochem kit (Cytoskeleton). Briefly, the pyrene actin was dissolved in G-buffer containing ATP at 0.4 mg/mL and incubated on ice for 1 h to depolymerize actin oligomers, followed by centrifuge at 14,000 rpm at 4°C for 30 min. The supernatant was transferred to a 96-well plate (200 μL per well). Meanwhile, the cells were lysed in actin-compatible buffer containing 20 mM HEPES, 20 mM NaCl, and protein inhibitors and subjected to centrifugation at 150,000 × g at 4°C for 1 h. 20 μL of the supernatant was added in each well containing G-actin stock. Then, actin polymerization buffer was added in the wells to start the reaction. Kinetic for over 120 cycles, 60 s interval time was set on Multiscan Spectrum (BioTek Synergy I) with the ex.350 nm and em.410 nm.

Statistical Analysis

All experiments were performed in triplicate, and numerical data were subject to independent sample t test for two groups and one-way ANOVA for three or more groups. The levels of significance were set at *p <0.05 and **p <0.01.

Declaration

Our study complies with the Declaration of Helsinki; the Animal Care Committee of Sunnybrook Research Institute has approved the research protocol and informed consent has been obtained from the subjects. Analysis of human heart tissues was approved by the Ethics Committee of Guangdong General Hospital.

SUPPLEMENTAL INFORMATION

Supplemental Information can be found online at https://doi.org/10.1016/j.ymthe.2020.12.004.

ACKNOWLEDGMENTS

This work was supported by grants from Canadian Institutes of Health Research (PJT-153105 and PJT-155962) to B.B.Y.

AUTHOR CONTRIBUTIONS

B.B.Y. supervised the project. N.W. and B.B.Y. designed the experiments. S.W. and J.X. collected patient samples. N.W., W.W.D., X.L., F.L., S.M., E.E., J.L., L.Z., K.Z., and A.A. performed the experiments. F.M.A. performed computational analysis of the interaction between circYap and the proteins. N.W. and B.B.Y. wrote the paper.

DECLARATION OF INTERESTS

The authors declare no competing interests.

REFERENCES

1. Thum, T., and Lorenzen, J.M. (2012). Cardiac fibrosis revisited by microRNA therapeutics. Circulation *126*, 800–802.
2. Camelliti, P., Borg, T.K., and Kohl, P. (2005). Structural and functional characterisation of cardiac fibroblasts. Cardiovasc. Res. *65*, 40–51.
3. Heinen, A., Nederlof, R., Panjwani, P., Spychala, A., Tschaidse, T., Reffelt, H., Boy, J., Raupach, A., Gödecke, S., Petzsch, P., et al. (2019). IGF1 Treatment Improves Cardiac Remodeling after Infarction by Targeting Myeloid Cells. Mol. Ther. *27*, 46–58.
4. Russo, I., Cavalera, M., Huang, S., Su, Y., Hanna, A., Chen, B., Shinde, A.V., Conway, S.J., Graff, J., and Frangogiannis, N.G. (2019). Protective Effects of Activated Myofibroblasts in the Pressure-Overloaded Myocardium Are Mediated Through Smad-Dependent Activation of a Matrix-Preserving Program. Circ. Res. *124*, 1214–1227.
5. Wu, J., Minikes, A.M., Gao, M., Bian, H., Li, Y., Stockwell, B.R., Chen, Z.N., and Jiang, X. (2019). Intercellular interaction dictates cancer cell ferroptosis via NF2-YAP signalling. Nature *572*, 402–406.
6. Hubaud, A., Regev, I., Mahadevan, L., and Pourquie, O. (2017). Excitable Dynamics and Yap-Dependent Mechanical Cues Drive the Segmentation Clock. Cell *171*, 668–682.e11.
7. Yang, Y., Ren, J., Sun, Y., Xue, Y., Zhang, Z., Gong, A., Wang, B., Zhong, Z., Cui, Z., Xi, Z., et al. (2018). A connexin43/YAP axis regulates astroglial-mesenchymal transition in hemoglobin induced astrocyte activation. Cell Death Differ. *25*, 1870–1884.
8. Xu, H., Zhou, S., Xia, H., Yu, H., Tang, Q., and Bi, F. (2019). MEK nuclear localization promotes YAP stability via sequestering β-TrCP in KRAS mutant cancer cells. Cell Death Differ. *26*, 2400–2415.
9. Zhou, Q., Li, L., Zhao, B., and Guan, K.L. (2015). The hippo pathway in heart development, regeneration, and diseases. Circ. Res. *116*, 1431–1447.
10. Morikawa, Y., Heallen, T., Leach, J., Xiao, Y., and Martin, J.F. (2017). Dystrophin-glycoprotein complex sequesters Yap to inhibit cardiomyocyte proliferation. Nature *547*, 227–231.
11. Gunthel, M., Barnett, P., and Christoffels, V.M. (2018). Development, Proliferation, and Growth of the Mammalian Heart. Mol. Ther. *26*, 1599–1609.
12. Wang, X., Ha, T., Liu, L., Hu, Y., Kao, R., Kalbfleisch, J., Williams, D., and Li, C. (2018). TLR3 Mediates Repair and Regeneration of Damaged Neonatal Heart through Glycolysis Dependent YAP1 Regulated miR-152 Expression. Cell Death Differ. *25*, 966–982.
13. Lin, Z., von Gise, A., Zhou, P., Gu, F., Ma, Q., Jiang, J., Yau, A.L., Buck, J.N., Gouin, K.A., van Gorp, P.R., et al. (2014). Cardiac-specific YAP activation improves cardiac function and survival in an experimental murine MI model. Circ. Res. *115*, 354–363.
14. Yuan, M., Tomlinson, V., Lara, R., Holliday, D., Chelala, C., Harada, T., Gangeswaran, R., Manson-Bishop, C., Smith, P., Danovi, S.A., et al. (2008). Yes-associated protein (YAP) functions as a tumor suppressor in breast. Cell Death Differ. *15*, 1752–1759.
15. Danovi, S.A., Rossi, M., Gudmundsdottir, K., Yuan, M., Melino, G., and Basu, S. (2008). Yes-associated protein (YAP) is a critical mediator of c-Jun-dependent apoptosis. Cell Death Differ. *15*, 217–219.
16. Salzman, J., Chen, R.E., Olsen, M.N., Wang, P.L., and Brown, P.O. (2013). Cell-type specific features of circular RNA expression. PLoS Genet. *9*, e1003777.
17. Wu, N., Yuan, Z., Du, K.Y., Fang, L., Lyu, J., Zhang, C., He, A., Eshaghi, E., Zeng, K., Ma, J., et al. (2019). Translation of yes-associated protein (YAP) was antagonized by its circular RNA via suppressing the assembly of the translation initiation machinery. Cell Death Differ. *26*, 2758–2773.
18. Du, W.W., Yang, W., Chen, Y., Wu, Z.K., Foster, F.S., Yang, Z., Li, X., and Yang, B.B. (2017). Foxo3 circular RNA promotes cardiac senescence by modulating multiple factors associated with stress and senescence responses. Eur. Heart J. *38*, 1402–1412.
19. Zeng, Y., Du, W.W., Wu, Y., Yang, Z., Awan, F.M., Li, X., Yang, W., Zhang, C., Yang, Q., Yee, A., et al. (2017). A Circular RNA Binds To and Activates AKT Phosphorylation and Nuclear Localization Reducing Apoptosis and Enhancing Cardiac Repair. Theranostics *7*, 3842–3855.
20. Gupta, S.K., Garg, A., Bär, C., Chatterjee, S., Foinquinos, A., Milting, H., Streckfuß-Bömeke, K., Fiedler, J., and Thum, T. (2018). Quaking Inhibits Doxorubicin-Mediated Cardiotoxicity Through Regulation of Cardiac Circular RNA Expression. Circ. Res. *122*, 246–254.
21. Li, H., Xu, J.D., Fang, X.H., Zhu, J.N., Yang, J., Pan, R., Yuan, S.J., Zeng, N., Yang, Z.Z., Yang, H., et al. (2019). Circular RNA circRNA_000203 aggravates cardiac hypertrophy via suppressing miR26b-5p and miR-140-3p binding to Gata4. Cardiovasc. Res. *116*, 1323–1334.
22. Garikipati, V.N.S., Verma, S.K., Cheng, Z., Liang, D., Truongcao, M.M., Cimini, M., Yue, Y., Huang, G., Wang, C., Benedict, C., et al. (2019). Circular RNA CircFndc3b modulates cardiac repair after myocardial infarction via FUS/VEGF-A axis. Nat. Commun. *10*, 4317.
23. Masedunskas, A., Appaduray, M.A., Lucas, C.A., Lastra Cagigas, M., Heydecker, M., Holliday, M., Meiring, J.C.M., Hook, J., Kee, A., White, M., et al. (2018). Parallel assembly of actin and tropomyosin, but not myosin II, during *de novo* actin filament formation in live mice. J. Cell Sci. *131*, jcs212654.
24. Lechuga, S., Baranwal, S., Li, C., Naydenov, N.G., Kuemmerle, J.F., Dugina, V., Chaponnier, C., and Ivanov, A.I. (2014). Loss of γ-cytoplasmic actin triggers myofibroblast transition of human epithelial cells. Mol. Biol. Cell *25*, 3133–3146.
25. Colpan, M., Ly, T., Grover, S., Tolkatchev, D., and Kostyukova, A.S. (2017). The cardiomyopathy-associated K15N mutation in tropomyosin alters actin filament pointed end dynamics. Arch. Biochem. Biophys. *630*, 18–26.
26. Du, W.W., Yang, W., Li, X., Fang, L., Wu, N., Li, F., Chen, Y., He, Q., Liu, E., Yang, Z., et al. (2020). The Circular RNA circSKA3 Binds Integrin beta1 to Induce Invadopodium Formation Enhancing Breast Cancer Invasion. Mol. Ther. *28*, 1287–1298.
27. Huang, W., Yang, Y., Wu, J., Niu, Y., Yao, Y., Zhang, J., Huang, X., Liang, S., Chen, R., Chen, S., and Guo, L. (2020). Circular RNA cESRP1 sensitises small cell lung cancer

cells to chemotherapy by sponging miR-93-5p to inhibit TGF-β signalling. Cell Death Differ. *27*, 1709–1727.

28. Zhao, W., Cui, Y., Liu, L., Qi, X., Liu, J., Ma, S., Hu, X., Zhang, Z., Wang, Y., Li, H., et al. (2020). Splicing factor derived circular RNA circUHRF1 accelerates oral squamous cell carcinoma tumorigenesis via feedback loop. Cell Death Differ. *27*, 919–933.
29. Lavenniah, A., Luu, T.D.A., Li, Y.P., Lim, T.B., Jiang, J., Ackers-Johnson, M., and Foo, R.S.Y. (2020). Engineered Circular RNA Sponges Act as miRNA Inhibitors to Attenuate Pressure Overload-Induced Cardiac Hypertrophy. Mol. Ther. *28*, 1506–1517.
30. Li, F., Yang, Q., He, A.T., and Yang, B.B. (2020). Circular RNAs in cancer: limitations in functional studies and diagnostic potential. Semin. Cancer Biol. Published online October 6, 2020. https://doi.org/10.1016/j.semcancer.2020.10.002.
31. Santer, L., Bar, C., and Thum, T. (2019). Circular RNAs: A Novel Class of Functional RNA Molecules with a Therapeutic Perspective. Mol. Ther. *27*, 1350–1363.
32. Sang, Y., Chen, B., Song, X., Li, Y., Liang, Y., Han, D., Zhang, N., Zhang, H., Liu, Y., Chen, T., et al. (2019). circRNA_0025202 Regulates Tamoxifen Sensitivity and Tumor Progression via Regulating the miR-182-5p/FOXO3a Axis in Breast Cancer. Mol. Ther. *27*, 1638–1652.
33. Wu, Y., Hong, Z., Xu, W., Chen, J., Wang, Q., Chen, J., Ni, W., Mei, Z., Xie, Z., Ma, Y., et al. (2020). Circular RNA circPDE4D Protects against Osteoarthritis by Binding to miR-103a-3p and Regulating FGF18. Mol. Ther. Published online September 5, 2020. https://doi.org/10.1016/j.ymthe.2020.09.002.
34. Frangogiannis, N.G. (2016). The Functional Pluralism of Fibroblasts in the Infarcted Myocardium. Circ. Res. *119*, 1049–1051.
35. Bai, F., Wang, L., and Kawai, M. (2013). A study of tropomyosin's role in cardiac function and disease using thin-filament reconstituted myocardium. J. Muscle Res. Cell Motil. *34*, 295–310.
36. Blanchoin, L., Pollard, T.D., and Hitchcock-DeGregori, S.E. (2001). Inhibition of the Arp2/3 complex-nucleated actin polymerization and branch formation by tropomyosin. Curr. Biol. *11*, 1300–1304.
37. Zhou, Y., Huang, X., Hecker, L., Kurundkar, D., Kurundkar, A., Liu, H., Jin, T.H., Desai, L., Bernard, K., and Thannickal, V.J. (2013). Inhibition of mechanosensitive signaling in myofibroblasts ameliorates experimental pulmonary fibrosis. J. Clin. Invest. *123*, 1096–1108.
38. Travers, J.G., Kamal, F.A., Robbins, J., Yutzey, K.E., and Blaxall, B.C. (2016). Cardiac Fibrosis: The Fibroblast Awakens. Circ. Res. *118*, 1021–1040.
39. Zhang, X., Javan, H., Li, L., Szucsik, A., Zhang, R., Deng, Y., and Selzman, C.H. (2013). A modified murine model for the study of reverse cardiac remodelling. Exp. Clin. Cardiol. *18*, e115–e117.
40. Du, W.W., Fang, L., Yang, W., Wu, N., Awan, F.M., Yang, Z., and Yang, B.B. (2017). Induction of tumor apoptosis through a circular RNA enhancing Foxo3 activity. Cell Death Differ. *24*, 357–370.
41. Yang, Z.G., Awan, F.M., Du, W.W., Zeng, Y., Lyu, J., Wu, D., Gupta, S., Yang, W., and Yang, B.B. (2017). The Circular RNA Interacts with STAT3, Increasing Its Nuclear Translocation and Wound Repair by Modulating Dnmt3a and miR-17 Function. Mol. Ther. *25*, 2062–2074.
42. Fang, L., Du, W.W., Awan, F.M., Dong, J., and Yang, B.B. (2019). The circular RNA circ-Ccnb1 dissociates Ccnb1/Cdk1 complex suppressing cell invasion and tumorigenesis. Cancer Lett. *459*, 216–226.
43. Tuszynska, I., Magnus, M., Jonak, K., Dawson, W., and Bujnicki, J.M. (2015). NPDock: a web server for protein-nucleic acid docking. Nucleic Acids Res. *43* (W1), W425–W430.
44. Pietal, M.J., Szostak, N., Rother, K.M., and Bujnicki, J.M. (2012). RNAmap2D - calculation, visualization and analysis of contact and distance maps for RNA and protein-RNA complex structures. BMC Bioinformatics *13*, 333.
45. Vangone, A., Spinelli, R., Scarano, V., Cavallo, L., and Oliva, R. (2011). COCOMAPS: a web application to analyze and visualize contacts at the interface of biomolecular complexes. Bioinformatics *27*, 2915–2916.

YMTHE, Volume *29*

Supplemental Information

YAP Circular RNA, circYap, Attenuates Cardiac Fibrosis via Binding with Tropomyosin-4 and Gamma-Actin Decreasing Actin Polymerization

Nan Wu, Jindong Xu, William W. Du, Xiangmin Li, Faryal Mehwish Awan, Feiya Li, Sema Misir, Esra Eshaghi, Juanjuan Lyu, Le Zhou, Kaixuan Zeng, Aisha Adil, Sheng Wang, and Burton B. Yang

Table S1. General information of patients

Parameters		Values
donation hearts(n)		21
Ages (Mean±SD)		36.87±15.17
Gender		
	Male	15
	Female	6
Etiology		
	trauma	11
	other non-heart disease	10
Cardiac hypertrophy (n)		104
Ages (Mean±SD)		23.21±24.88
Gender		
	Male	63
	Female	41
Etiology		
	Heart failure	25
	Aortic stenosis	4
	Mitral stenosis	3
	Hypertrophic cardiomyopathy	8
	Tetralogy of Fallot	64

Table S2. Primer information.

Name		sequence
circYap junction	Forward:	5’-GCAAGAACTGCTTCGGCAGGTCCT-3’
	Reverse:	5’-GTTTATATAGTAAATTTCTCCATC-3’
circYap non-junction	Forward:	5'-GCAGCAACTGCAGATGGAGAAGGAG-3'
	Reverse:	5'-GGGTCTAGCCAAGAGGTGGTCTTG-3'
circYap linear precursor	Forward:	5'-TCTTCCTGATGGATGGGAAC-3'
	Reverse:	5'-GGCTGTTTCACTGGAGCACT-3'
Yap mRNA	Forward:	5’-CCGTGCCCATGAGGCTCCGGAAGC-3’
	Reverse:	5’-GGGTGTAGCTGCTGGGCCAGAGAC-3’
U6	Forward:	5'-GTGCTCGCTTCGGCAGCACATA-3'
	Reverse:	5'-TGGAACGCTTCACGAATTTGCG-3'
Collagen-I	Forward:	5'-GAATGGAGATGATGGGGAAGCTGG-3'
	Reverse:	5’-CATCTCCTTTGGCACCATCCAAAC-3'
Collagen-III	Forward:	5’-GTAAAGAAGTCTCTGAAGCTGATG-3'
	Reverse:	5’-GCGATATCTATGATGGGTAGTCTC-3'
TGF-β1	Forward:	5'-GAGCCTGAGGCCGACTACTA-3'
	Reverse:	5'-GGGTTCAGGTACCGCTTCTC-3'
CTGF	Forward:	5'-CAAGGGCCTCTTCTGTGACT-3'
	Reverse:	5'-ACGTGCACTGGTACTTGCAG-3'
NGF	Forward:	5'-AGGGAGCAGCTTTCTATCCTG-3'
	Reverse:	5'-GGC AGT GTC AAG GGA ATG C-3'
TNF-α	Forward:	5'-CCCAGGGACCTCTCTCTAATCA-3’
	Reverse:	5'-AGCTGCCCCTCAGCTTGAG-3’
TPM4	Forward:	5'-AAGTGTTGGGCAACAAAAGG-3'
	Reverse:	5'-CTTGGCAAAGTCAAGCTTCC-3'
ACTG	Forward:	5'-ATGTTGCCCTGGATTTTGAG-3'
	Reverse:	5'-AGGAAGGAAGGCTGGAAGAG-3'

Table S3. Mass spectrophotometry showing interaction of circYAP with proteins

Accession No.	Alternate ID	mol wt	vector	circYap
O75367 (+2)	H2AFY	40 kDa	8.7988	5.0694
P08238	HSP90AB1	83 kDa	14.665	5.0694
P0CG39	POTEJ	117 kDa	69.413	0
P11940 (+1)	PABPC1	71 kDa	14.665	4.0555
P14625	HSP90B1	92 kDa	5.8659	2.0278
P16989 (+2)	YBX3	40 kDa	5.8659	2.0278
P24534	EEF1B2	25 kDa	2.9329	1.0139
P27348	YWHAQ	28 kDa	2.9329	0
P27482	CALML3	17 kDa	0	5.0694 ←
P29692 (+1)	EEF1D	31 kDa	2.9329	0
P31946 (+1)	YWHAB	28 kDa	5.8659	0
P38159 (+1)	RBMX	42 kDa	5.8659	6.0833
P47756	CAPZB	31 kDa	0	18.25 ←
P51991 (+1)	HNRNPA3	40 kDa	14.665	6.0833
P62258	YWHAE	29 kDa	3.9106	0
P62807	HIST1H2BC	14 kDa	0	97.332 ←
P62995	TRA2B	34 kDa	0	2.0278 ←
P63104	YWHAZ	28 kDa	6.8435	3.0416
P63261	ACTG1	42 kDa	0	191.62 ←
P67936	TPM4	29 kDa	0	128.76 ←
P68363 (+1)	TUBA1B	50 kDa	9.7765	4.0555
P68366 (+1)	TUBA4A	50 kDa	6.8435	0
P68371	TUBB4B	50 kDa	16.62	6.0833
P68431	HIST1H3A	15 kDa	24.441	0
P84243	H3F3A	15 kDa	0	30.416 ←
Q04917	YWHAH	28 kDa	4.8882	0
Q13595 (+2)	TRA2A	33 kDa	2.9329	1.0139
Q13885	TUBB2A	50 kDa	12.709	7.0971
Q15233 (+1)	NONO	54 kDa	0	6.0833 ←
Q32MZ4-2 (+1)	LRRFIP1	86 kDa	0	4.0555 ←
Q7Z794	KRT77	62 kDa	0	2.0278 ←
Q8WXF1 (+1)	PSPC1	59 kDa	4.8882	2.0278
Q96E39	RBMXL1	42 kDa	0	4.0555 ←
Q9H361	PABPC3	70 kDa	7.8212	0
Q9NYF8 (+2)	BCLAF1	106 kDa	0	2.0278 ←
Q9P0M6	H2AFY2	40 kDa	6.8435	3.0416

Arrows show proteins potentially interacting with circYap.

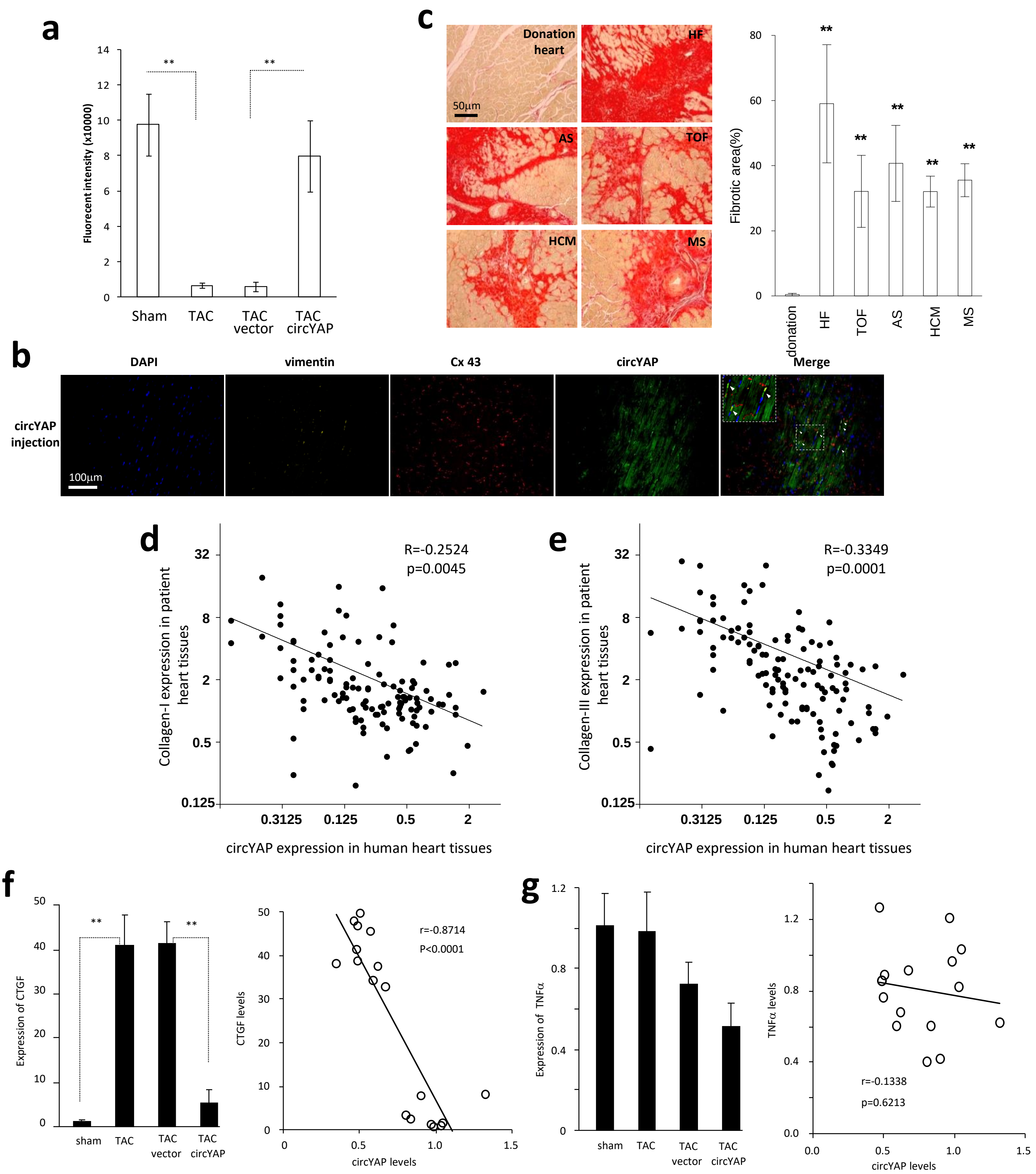

Fig S1. The circYap and fibrosis in heart.

(a) Quantitation of fluorescence in situ hybridization staining. n=4. **p<0.01.

(b) Co-localization of circYap with cardiac fibroblasts (marked with vimentin) and cardiomyocytes (marked with connexin) in mouse heart with TAC and circYap plasmid injection. Blue: DAPI; Yellow: Vimentin; Red, Cx43; Green circYap.

(c) Representative images of sirius red staining in human heart with or without cardiac hypertrophy. n=3.

(d-e) Correlation of circYap expression with collagen-I and collagen-II levels in human heart tissues. n=125

(f) Left: Expression of CTGF in mouse heart tissues of sham, TAC, TAC+vector and TAC+circYap mice. n=5. **p<0.01. Right: Correlation between CTGF and circYap levels n=18.

(g) Left: Expression of TNFα in mouse heart tissues of sham, TAC, TAC+vector and TAC+circYap mice. n=5. **p<0.01. Right: Correlation between TNFα and circYap levels n=16.

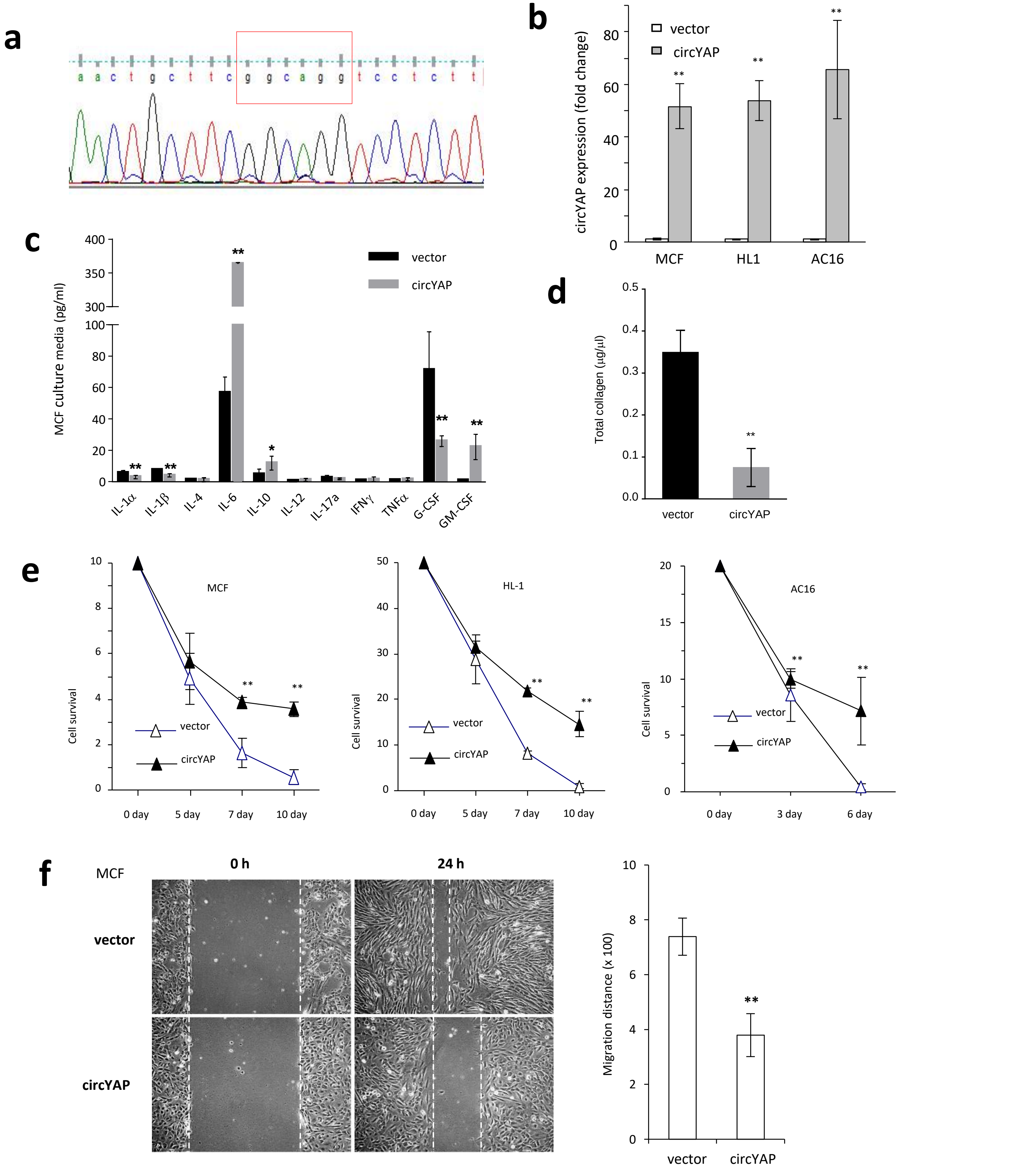

Fig S2. The role of circYap in heart cell function.

(a) Sanger sequence of the back-splice junction of ectopic human circYap.

(b) Ectopic expression of circYap in mouse cardiac fibroblasts (MCF), cardiomyocytes (HL-1) and human cardiomyocytes (AC16). n=4. **p<0.01.

(c) Cytokines that MCF secreted in cell culture media after TGF-β (1ng/ml) treated for 24h were detected using Qiagen multi-Analyte ELISArray Kit. n=6. **p<0.01, *p<0.05.

(d) Total collagen levels in cell culture media after TGF-β (1ng/ml) treated for 24h were detected using Abcam total collagen assay kit. n=5. **p<0.01.

(d) Overexpression of circYap constructs in MCF, HL-1 and AC16 cells increased cell survival. n=3. **p<0.01

(b) Effect of circYap on MCF migration. n=4. **p<0.01.

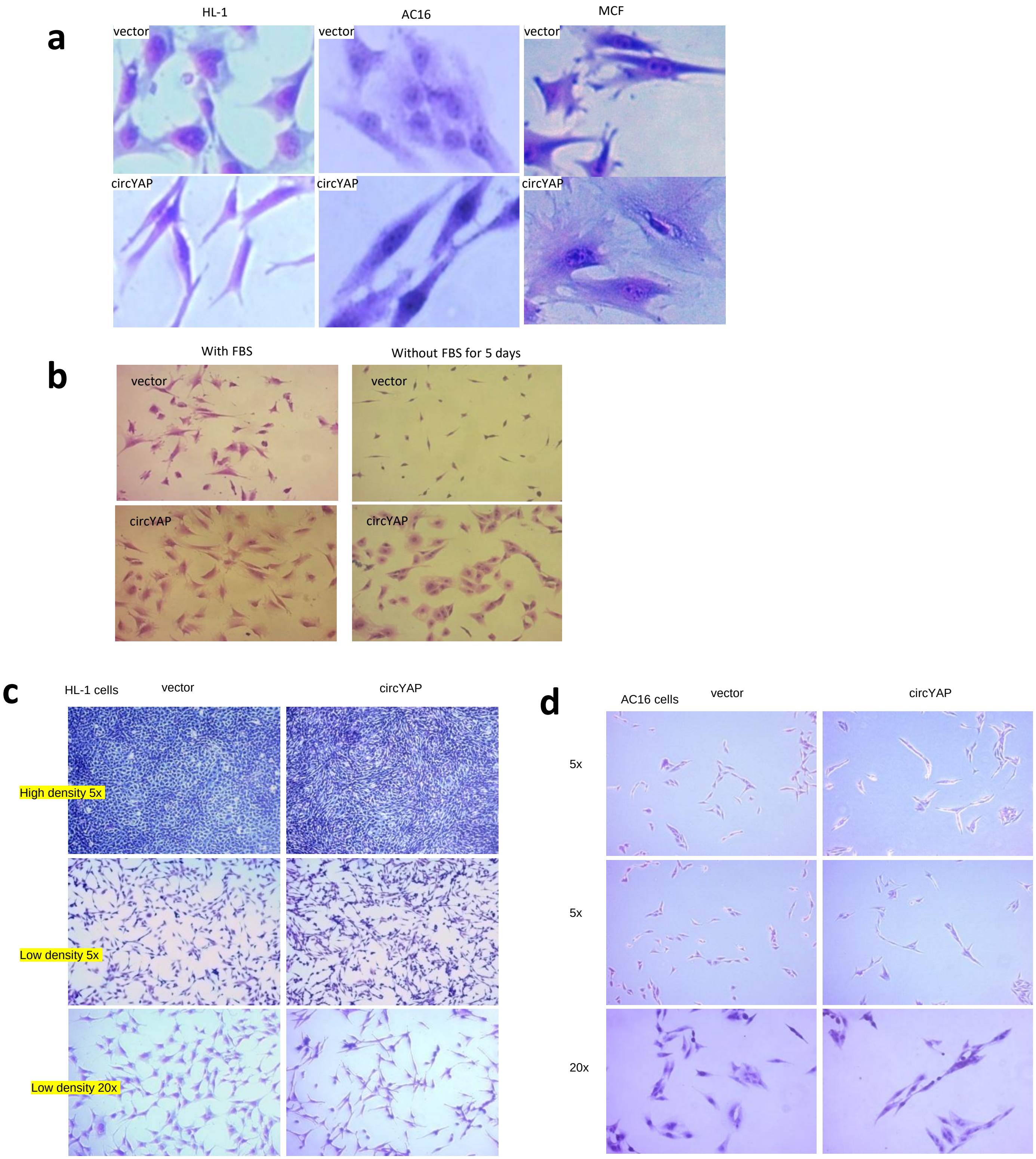

Fig S3. The role of circYap in change of cell morphology.

(a) Overexpression of circYap construct decreased cell spreading in HL-1 and AC16 cells, but increased cell spreading in MCF cells.

(b-d) Morphology changes after overexpression of circYap in HL-1, AC16 and MCF cells (large view).

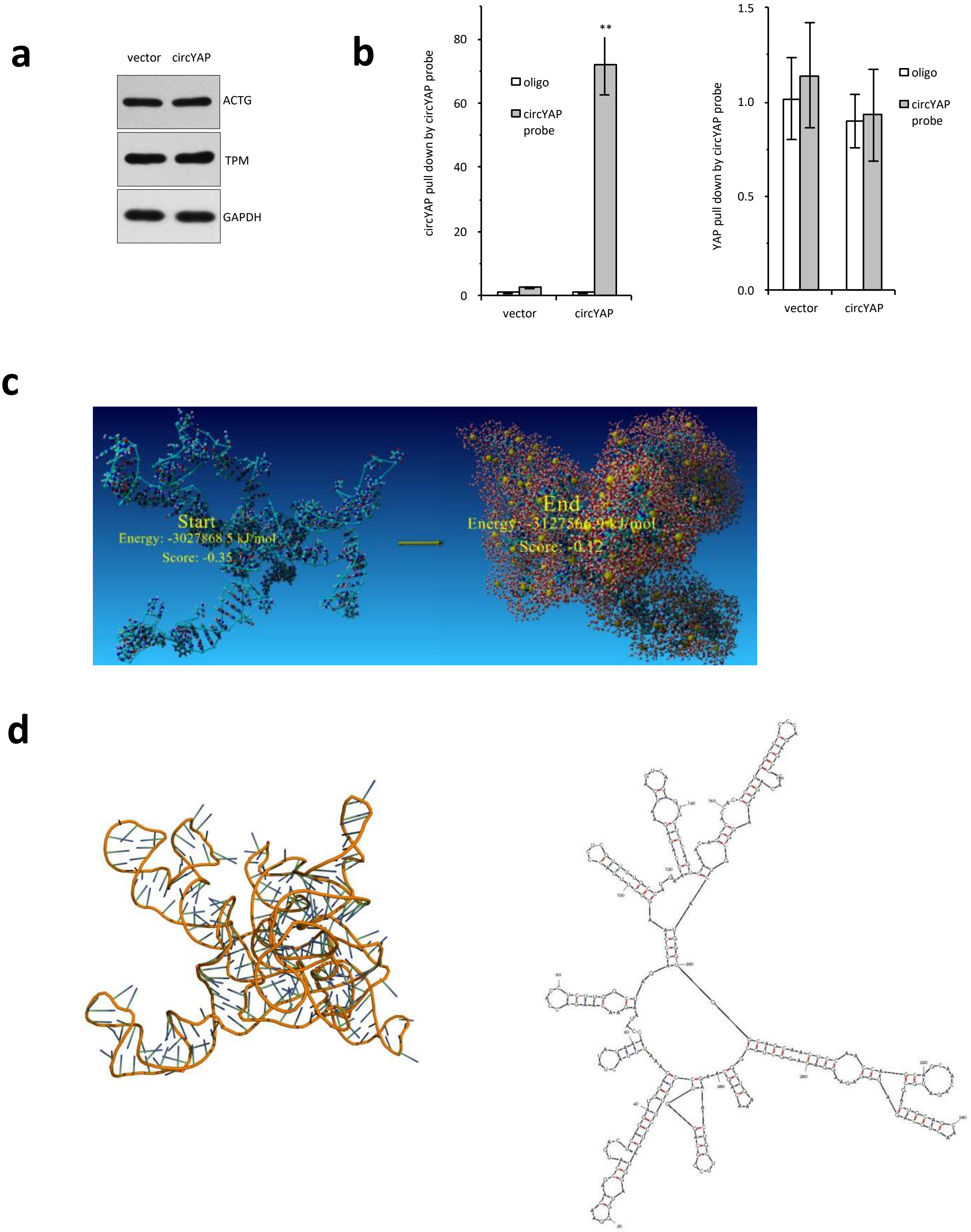

Fig S4. Two dimensional structure of circYap.
(a) Overexpression of circYap did not change levels of ACTG and TPM4.
(b) circYap probe could pull down circYap (left) but had no effect on pulling down linear YAP mRNA.
(c) YASARA representation of energy minimized 3D circ-YAP1 RNA.
(d) Two- and three-dimensional structures of circYap sequence.

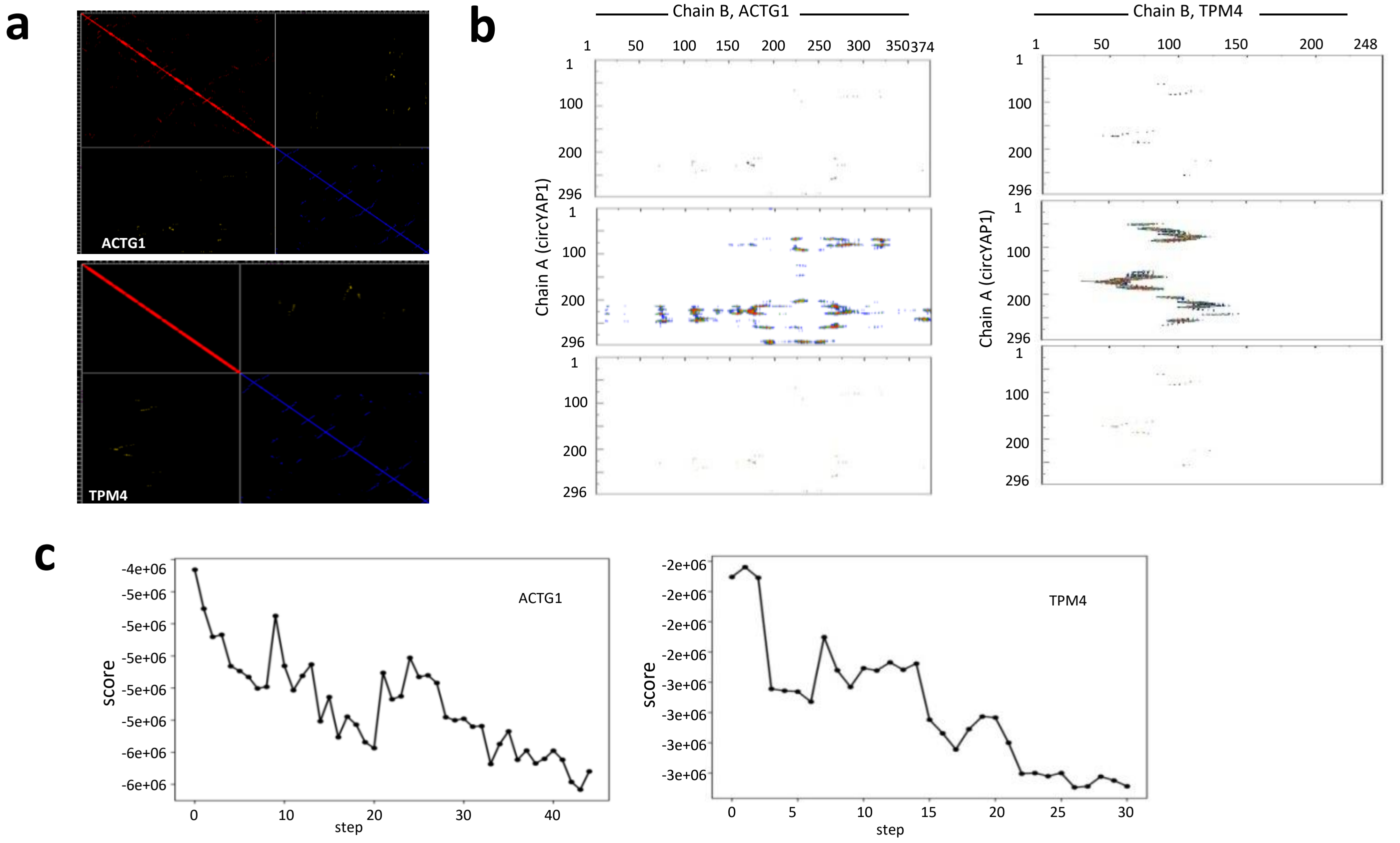

d

His-tagged ACTG construct with circYAP binding sites mutation
gccgccaccatggaagaagagatcgccgcgctggtcattgacaatggctccggcatgtgcaaagctggttttgctggggacgacgctccccgagccgtgtttccttccatcgtcgggcgccccagacaccagggcgtcatggtgggcatgggccagaaggactcctacgtgggcgacgaggcccagagcaagcgtggcatcctgaccctgaagtaccccattgagcatggcatcgtcaccaactgggacgacatggagaagatctggcaccacaccttctacaacgagctgcgcgtggccccggaggagcacccagtgctgctgaccgaggcccccggcggaggcggaggcaacagagagaagatgactcagattatgtttgagaccttcaacaccccggccatgtacgtggccatccaggccgtgctgtccctctacgcctctgggcgcaccactggcattgtcatggactctggagacggggtcacccacacggtgcccatctacgagggctacgccggcggcggagccggcggtggtggtggactggctggccgggacctgaccgactacctcatgaagatcctcactgagcgaggctacagcttcaccaccacggccgagcgggaaatcgtgcgcgacatcaaggagaagctgtgctacgtcgccctggacttcgagcaggagatggccaccgccgcatcctcctcttctctggagaagagctacgagctgcccgatggccaggtcatcaccattggcaatgagcggttccggtgtccggaggcgctgttccagcctggcggtctgggtggaggaggtggcggcatccacggaggcaccttcggaggtatcggtaagtgtgacgtggacatccgcaaagacctgtacgccaacacggtgctgtcgggcggcaccaccatgtacccgggcattgccgacaggatgcagaaggagatcaccgccctggcgcccagcaccatgaagatcaagatcatcgcaccccccagagcgcaagtactcggtgtggatcggtggctccatcctggcctcactgtccaccttccagcagatgtggattagcaagcaggagtacgacgagtcgggcccctccatcgtccaccgcaaatgcttccaccatcaccatcaccatcactaa

His-tagged TPM4 construct with circYAP binding sites mutation
gccgccaccatggccggcctcaactccctggaggcggtgaaacgcaagatccaggccctgcagcagcaggcggacgaggcggaagaccgcgcgcagggcctgcagcgggagctggacggcgagcgcgagcggcgcgagaaagctgaaggtgatgtggccggcggtggaggaggcggtcagctcggtgaggagggtggtgacagggctggagaacgaggtggcacggccggtggaaagggtggagaggcagaaaaagctgcagatgagagtgagagaggaatgaaggtgatagaaaaccgggccatgaaggatgaggagaagatggagattcaggagatgcagctcaaagaggccaagcacattgcggaagaggctgaccgcaaatacgaggaggtagctcgtaagctggtcatcctggagggtgagctggagagggcagaggagcgtgcggaggtgtctgaactaaaatgtggtgacctggaagaagaactcaagaatgttactaacaatctgaaatctctggaggctgcatctgaaaagtattctgaaaaggaggacaaatatgaagaagaaattaaacttctgtctgacaaactgaaagaggctgagacccgtgctgaatttgcagagagaacggttgcaaaactggaaaagacaattgatgacctggaagagaaacttgcccaggccaaagaagagaacgtgggcttacatcagacactggatcagacactaaacgaacttaactgtatacaccatcaccatcaccatcactaa

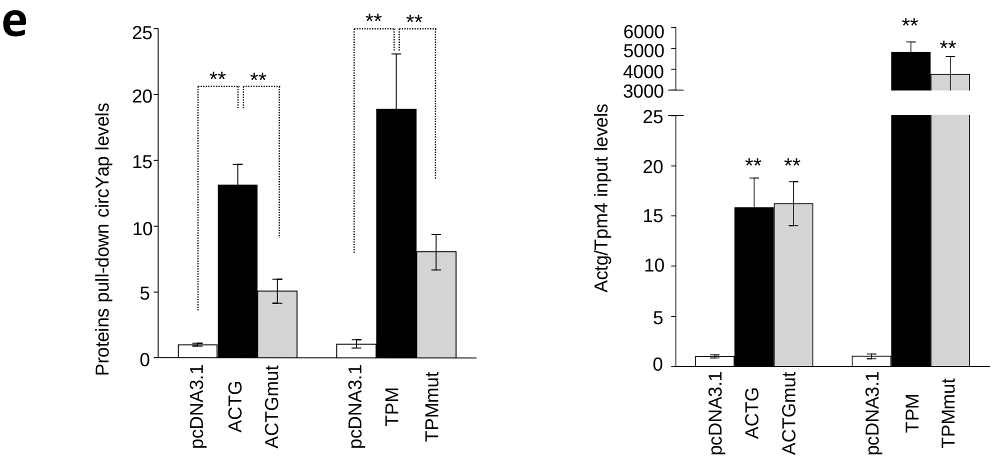

Fig S5. Computational analysis of circYap interacting with ACTG and TPM4.

(a) The contact map in the binding residues between circYap and ACTG or TPM4.

(b) The residue-level resolution contact maps in the binding residues between circYap and ACTG or TPM4.

(c) Refinement of the best docked circYap-ACTG model and circYap-TPM4 model showing MC score vs. steps of simulation.

(d) Plasmids that could be translated to His-tagged ACTG and TPM4 protein with or without circYap binding sites mutation (ACTGmut and TPM4mut) were transfected to circYap-overexpressed MCF cells. Ni-NTA beads were used to precipitate His-tagged ACTG and TPM4 and their bound circYap. n=6. **p<0.01.

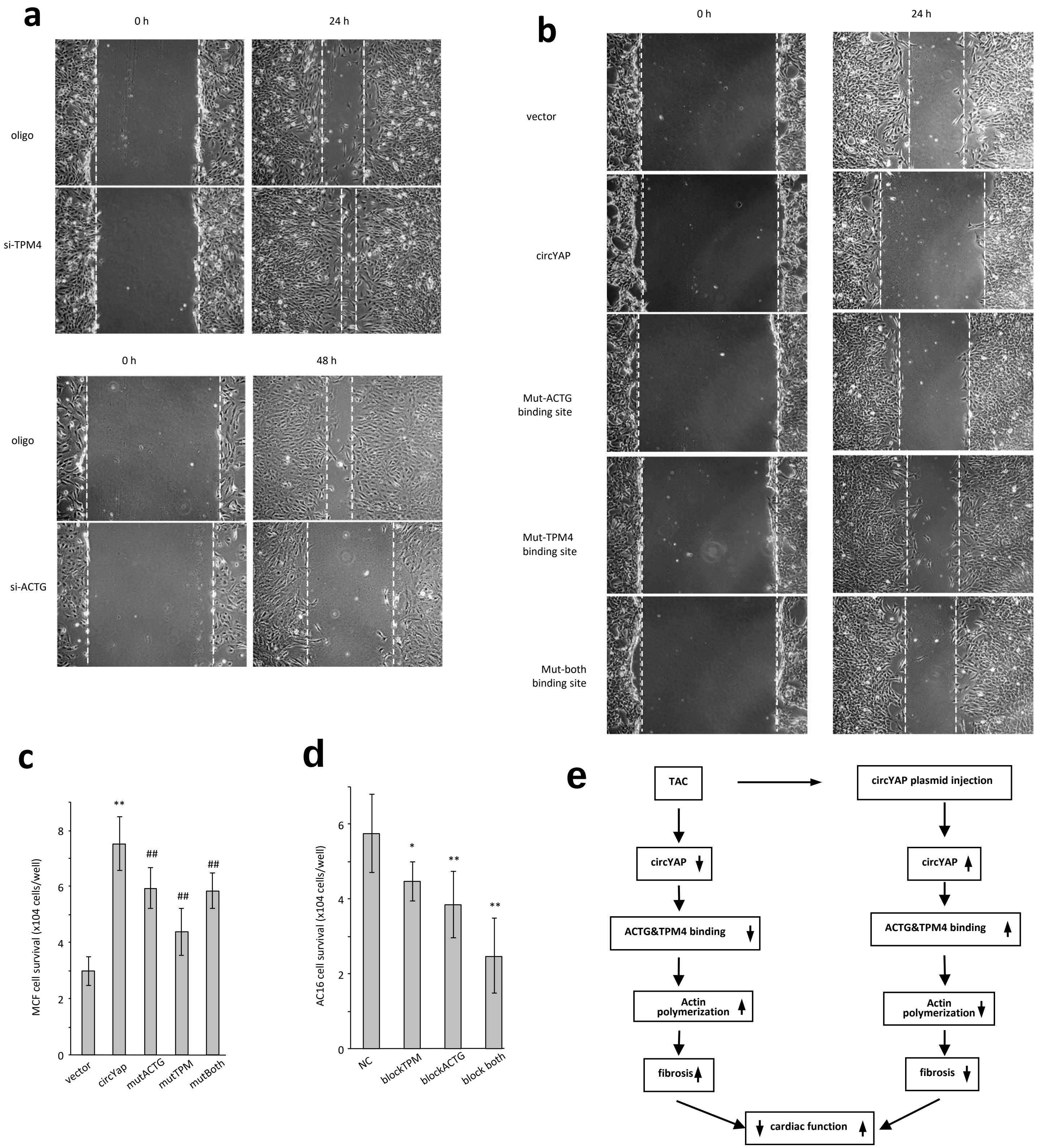

Fig S6. The role of circYap in cell migration

(a) Silencing TPM4 increased cell migration while silencing ACTG decreased cell migration.

(b) Overexpression of circYap decelerated cell migration while mutation of the binding sites in circYap abolished such effect.

(c) The survival rates upon serum deprivation in vector control, circYap, and plasmids containing mutations in the binding sites with ACTG and TPM4. n=6. $^{**}p<0.01$ vs vector, $^{\#\#}p<0.01$ vs circYap

(d) The survival rates of AC16 cells transfected with the blocking oligos upon serum deprivation. n=6. $^{**}p<0.01$ vs vector, $^{\#\#}p<0.01$ vs circYap.

(e) Diagram showing the mechanisms of circYap effects on heart fibrosis.
